# A Coding Method for Efficient Subgraph Querying on Vertex- and Edge-Labeled Graphs

**DOI:** 10.1371/journal.pone.0097178

**Published:** 2014-05-22

**Authors:** Lei Zhu, Qinbao Song, Yuchen Guo, Lei Du, Xiaoyan Zhu, Guangtao Wang

**Affiliations:** Department of Computer Science and Technology, Xi’an Jiaotong University, Xi’an, China; Swiss Institute of Bioinformatics, Switzerland

## Abstract

Labeled graphs are widely used to model complex data in many domains, so subgraph querying has been attracting more and more attention from researchers around the world. Unfortunately, subgraph querying is very time consuming since it involves subgraph isomorphism testing that is known to be an NP-complete problem. In this paper, we propose a novel coding method for subgraph querying that is based on Laplacian spectrum and the number of walks. Our method follows the filtering-and-verification framework and works well on graph databases with frequent updates. We also propose novel two-step filtering conditions that can filter out most false positives and prove that the two-step filtering conditions satisfy the no-false-negative requirement (no dismissal in answers). Extensive experiments on both real and synthetic graphs show that, compared with six existing counterpart methods, our method can effectively improve the efficiency of subgraph querying.

## Introduction

Labeled graphs, which include both vertex- and edge-labeling, have been widely used to model complicated structures and schemaless data in many domains such as social network [1], [2], chemistry [3], [4], image analysis [5], [6], and XML documents [7], [8]. This triggers the needs for effective graph pattern discovery, and the most compelling one is subgraph querying.

**Figure 1:**
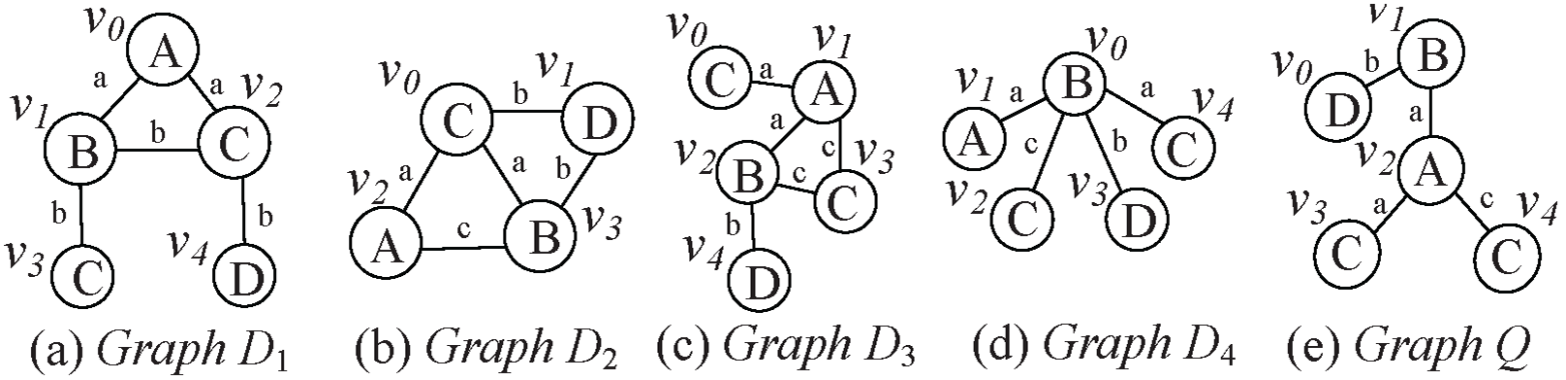
An Example of Subgraph Query. Four labeled graphs (a) 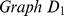, (b) 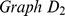, (c) 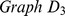, and (d) 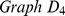 compose the database, and (e) 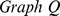 is a query graph.

The subgraph query problem is to retrieve all the supergraphs of a given graph from a graph database. It can be defined as follows: for a large graph database 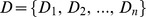 and a query graph *Q*, subgraph query is to find all the graphs 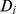 (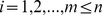) such that *Q* is a subgraph of 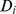. Fig. 1 shows an example of subgraph query, where the graph database consists of graphs 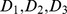 and 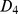, and *Q* is the query graph. Obviously, only graph 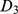 contains *Q*.

However, it is intractable to find all supergraphs of a query graph from a large graph database, since subgraph query must conduct subgraph isomorphism testing, which is a NP-complete problem [9], [10]. In order to address this problem, the filtering-and-verification framework is commonly adopted by most existing methods. These methods first extract some “useful” graph features and build indexes for them; then, in the filtering phase, they traverse the indexes to prune most false positives and generate the candidate graph set; after that, in the verification phase, they validate the candidate graphs with subgraph isomorphism testing and obtain the answer set.

Among the existing subgraph query methods, some of them, such as GraphGrep [11], gIndex [12], FG-Index [13], Treepi [14], Tree+delta [15] and SwiftIndex [16], build the inverted indexes for features that are substructures extracted from graph databases. The path extracted by GraphGrep is too simple and leads to low filtering efficiency [12]. Other methods have to re-mine frequent substructures and re-build indexes from scratch for the databases with frequent updates, so are quite time consuming [17].

Closure-tree method [18] uses clustering techniques to build indexes. It clusters a set of graphs into several groups, and each group is referred to as a graph closure. The graph closures are then used as nodes to build an index tree. By traversing the index tree, this method finds out a disqualified node via the pseudo subgraph isomorphism testing, and all graphs contained in this node are pruned. As Closure-tree uses the expensive pseudo subgraph isomorphism testing to filter out false positives, it costs too much time in the filtering phase [19], [20].

There are subgraph query methods, for example GCoding [17] and LsGCoding [21], which use graph coding methods to build indexes. These methods extract high-quality features from graphs, and map them into numerical space to generate graph codes. For a specific feature, if its corresponding code in a query graph is greater than that of a graph 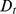, the query graph is not a subgraph of graph 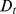. So, 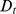 can be filtered as a false positive. According to this logic, these methods build indexes based on codes to filter out false positives. Moreover, these methods individually encode each graph. When the graph database is updated with lots of insertions and deletions, these methods do not need to re-compute graph codes and re-build the indexes from scratch. However, the subtree extracted by GCoding represents partial structure, which degrades its filtering efficiency; and Laplacian matrix used in LsGCoding only represents graphs with unlabeled edges, which makes LsGCoding can only process graphs with unlabeled edges.

In order to conduct subgraph query on labeled graphs, we propose a novel Laplacian spectrum and the number of walks based Graph Coding (LnGCoding) method by extending LsGCoding method. The extended method LnGCoding can generate new codes, which include the vertex labels and the labels of adjacent edges consisting of the labels of edges, Laplacian spectrum, and the number of walks. These are new features and not contained in the codes of LsGCoding. Based on the new codes, a novel index tree and a novel two-step filtering conditions are proposed in LnGCoding. Since the codes contain more information, LnGCoding not only conducts subgraph querying on labeled graphs, but also effectively filters out most false positives. Moreover, it works well in the databases with frequent updates. Extensive experiments on both real and synthetic data show that our proposed method LnGCoding can improve the efficiency of subgraph query, especially on dense graphs with labeled edges.

## Methods

In this section, we present the novel coding method and its application in subgraph query. At first, we introduce the definitions of vertex and graph codes, the properties of graph features, and the coding method based on these graph features. Then, we state the index building method based on the novel graph codes, and provide the filtering conditions generation method. Finally, based on the indexes and the filtering conditions, we present the filtering-and-verification framework for subgraph query. Note that, a labeled graph is abbreviated to a graph in the rest of this paper.

### Definitions of Vertex and Graph Codes

In our method, the vertex and graph codes are based on Laplacian spectrum and the number of walks. Therefore, we first give the definitions of adjacency matrix, Laplacian matrix and spectrum, walk and path. Then, based on these definitions, we define the vertex and graph codes.

**Definition 1** *(Adjacency Matrix of Graph). Given a graph G with 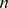 vertices, its adjacency matrix is defined as 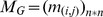, where*

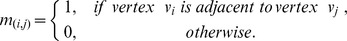



**Definition 2** *(Laplacian Matrix and Laplacian Spectrum of Graph). Given a graph G with 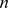 vertices, its Laplacian Matrix is defined as 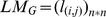, where*

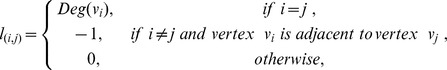

*and* 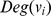 *is the degree of vertex* 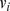.

*All eigenvalues of 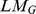 are called graph G’s Laplacian Spectrum.*

**Definition 3** *(Walk and Path). A walk in graph G consists of a pair (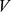, 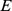) of sequences, where 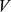 is a vertex sequence: 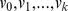, and 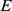 is an edge sequence: 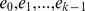. For 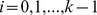, each successive pair 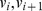 of a vertex is adjacent in G, and edge 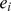 has 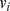 and 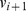 as terminal vertices.*

*A path is a walk with no repeated edges.*

*For a path, no edge occurs more than once in the edge sequence. This is different from a walk. The length 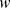 of a walk (or path) is the number of edges which occur in the walk (or path).*

**Definition 4** *(Vertex Code). Given a graph G and a vertex 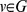, the vertex code 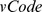 of v is a quadruple:*

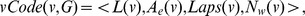

*where 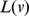 is a length-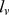 (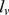 is a integer) counter string that denotes the vertex label of v, 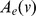 is a length-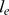 (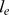 is a integer) counter string that denotes the labels of adjacent edges from v, 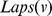 is the Laplacian spectrum of neighborhood graph of v, and 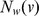 is a length-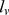 counter string that denotes the number of walks of length W (W is an integer) from v. Note that, the counter string is an array of multi-digit counters, where each element counts the occurrences of the specified vertices/edges/walks in a graph; And the adjacent edge labels of v are two-tuples, consisting of the labels of edges and the label of the terminal vertex that is on the same edge as v.*

**Figure 2:**
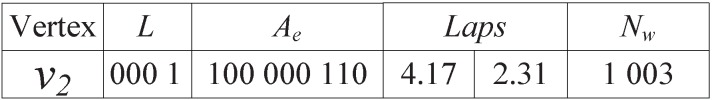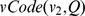. The vertex 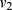 and graph 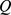 both occurred in Figure 1.

Fig. 2 shows 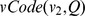 of vertex 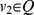, which occurred in Fig. 1. For the sake of convenience, the first two largest Laplacian eigenvalues are used to denote the Laplacian spectrum of each vertex, and the length 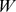 of walks is set to 2.

**Definition 5** *(Graph Code). Given a graph G with n vertices, and that vertex code of vertex 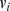 is denoted as 
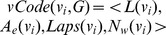
, for 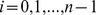. The graph code 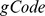 of G is defined as a quadruple:*

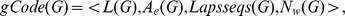

*where* 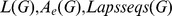 *and* 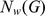 *are defined as follows:*

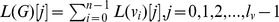
;

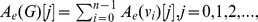
 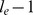;

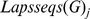
 = *The ranked Laplacian spectrum 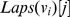 of all vertices with non-ascending order,* 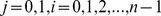;

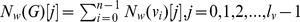
.

**Figure 3:**
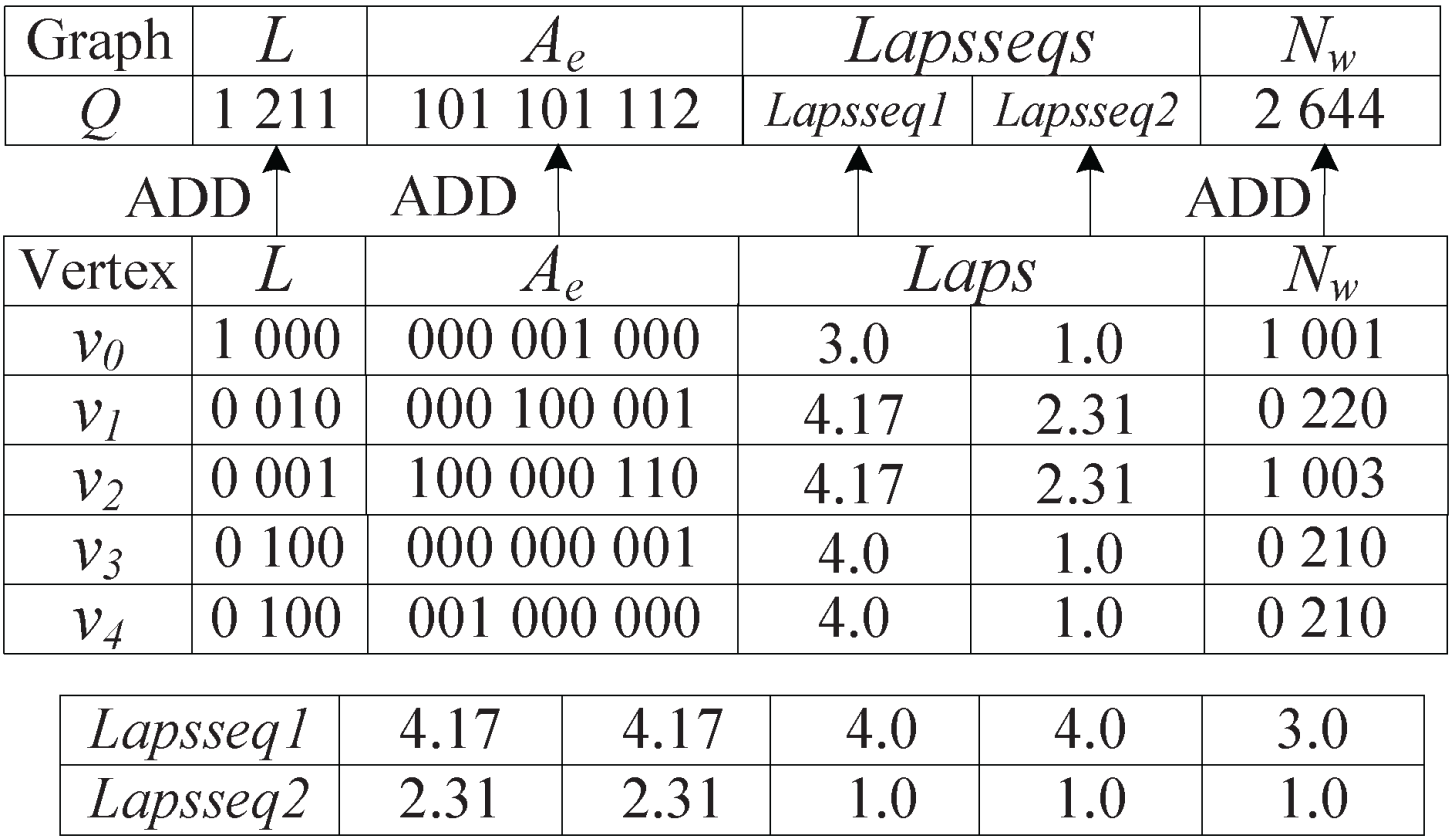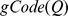. All of 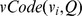 (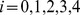) are combined to the 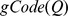.

Fig. 3 shows the graph code of graph *Q*. Where 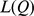, 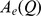 and 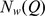 are generated by combining 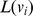, 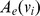 and 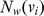 codes of all vertices 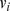 (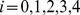) with the element-wise ADD operation. Here, the element-wise ADD operation of counter strings 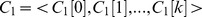 and 
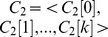
 is defined as 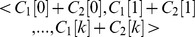, and 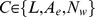. For 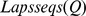, we rank all the corresponding eigenvalues 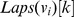 in the non-ascending order, and the results *Lapsseq1* and *Lapsseq2* are its Laplacian spectrum sequences *Lapsseqs*.

### The Properties of Graph Features

In our coding method, the codes consist of the following features: i) the labels of vertices and adjacent edges, ii) Laplacian spectrum, and iii) the number of walks. Since these features have the following properties, we can use them to efficiently and effectively filter out false positives.

#### The labels of vertices and adjacent edges

This is the first graph feature in our proposed method. As we all know, for each vertex (or edge) of a graph, there exists a corresponding vertex (or edge) in its supergraph. Based on this, we have the lemma as follows.

**Lemma 1** *Let graph 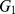 be a subgraph of graph 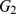, for a specific label l, the number of vertices (or edges) with label l in 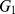 is not more than the number of vertices (or edges) with label l in 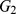.*

Applying the converse-negative proposition of Lemma 1 to vertices and graphs, we have the following corollaries.

**Corollary 1** *Given two graphs 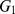 and 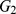, and the two vertices 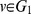 and 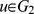 have the same vertex label. If there exists a specific adjacent edge label l, and the number of adjacent edges with label l of vertex v is more than the number of adjacent edges with label l of u, then u is not a corresponding vertex of v.*

**Corollary 2** *Given two graphs 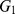 and 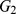, if there exists a specific label l, and the number of vertices (or adjacent edges) with label l in 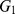 is more than the number of vertices (or adjacent edges) with label l in 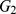, then 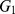 is not a subgraph of 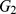.*

#### Laplacian spectrum

We choose Laplacian spectrum as the second feature, since there exists a relationship between the Laplacian spectrum of a graph and the Laplacian spectra of its subgraphs, and this relationship can be used to efficiently filter out false positives.

In order to prove there does exist the relationship, we first introduce 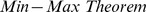 [22] as follows.

**Theorem 1** *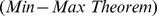. Given a real symmetric matrix 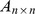

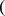

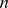
 is an integer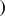, and its eigenvalues are 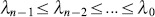. Then the eigenvalues of matrix A are represented as follows:*

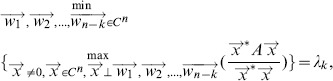



*and*

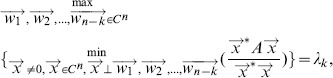

*where 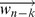

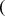

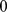

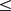

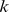

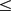

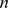
-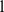

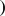
 and 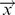 are n-dimensional vectors, and 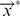 is the transposition of* 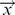.

In Algebraic Graph Theory [23], according to the properties of Laplacian matrices, the Laplacian matrix of a graph is a real symmetric matrix, and each eigenvalue of a Laplacian matrix is not less than zero. Thus, the Laplacian matrix of a graph is a real symmetric positive semidominant matrix. Applying 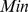-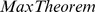 to the positive semidominant matrixes, we can have the following corollary.

**Corollary 3** *Let 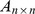 and 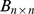 be two real symmetric matrices, and their eigenvalues be 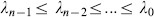 and 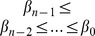, respectively. If matrix 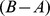 is a positive semidominant matrix, then for each 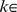
*{*
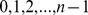
*}*, 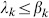 holds.*

*Proof.* According to 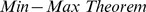, the eigenvalues of 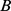 can be represented as follows: 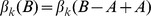


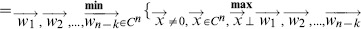


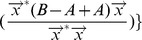


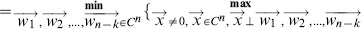


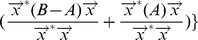


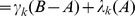
where 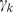 is the 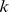-th eigenvalue of matrix 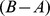. As matrix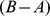 is a positive semidominant matrix, the 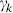 is not less than zero. Thus, we have



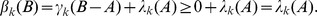



According to Corollary 3, if two real symmetric matrices *A* and *B* satisfy that matrix 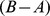 is a real symmetric positive semidominant matrix, the eigenvalues of *B* is not less than that of *A*. Since the Laplacian matrix of each graph is a real symmetric positive semidominant matrix, we can apply Corollary 3 to a graph and its subgraphs, and thus have the following theorem.

**Theorem 2** *For graph 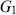 with m vertices and graph 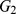 with 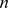

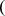
m
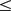
n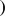 vertices, suppose 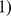 the matrix 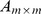 is the Laplacian matrix of 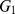, and 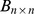 is the Laplacian matrix of 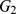; 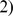 the eigenvalues of matrix A are 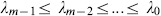, and the eigenvalues of matrix B are 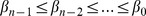. If 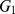 is a subgraph of 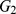, then for each 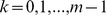, Laplacian spectra of 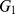 and 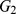 satisfy 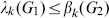.*

*Proof*. (sketch) Since 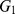 is a subgraph of 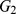, we can first generate a new graph 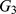 by adding 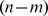 vertices to graph 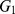, and these vertices occur in 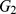 but not in 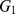; and then achieve the 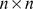 Laplacian matrix 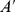 of 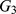 by adding 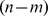 elements ″0″ to the 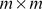 matrix 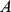. This ensures that 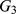 is also a subgraph of 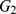, and 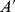 have the same non-zero eigenvalues as *A*. Meanwhile, we generate a new graph 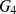 by removing the edges in 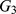 from 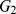. And Laplacian matrix of 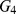 can be denoted as matrix 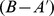. For a given graph, its Laplacian matrix is a real symmetric positive semidominant matrix. Thus, Laplacian matrices 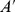, 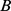 and 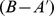 are all real symmetric positive semidominant matrices. According to Corollary 3, for each 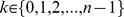, we have 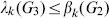. Furthermore, for each 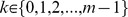, 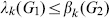 holds.

Applying the converse-negative proposition of Theorem 2 to Laplacian spectra of graphs, we have a useful corollary as follows.

**Corollary 4** *Given two graphs 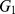 with m vertices and 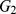 with n vertices (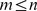), Laplacian spectrum of 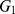 is 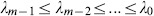, and Laplacian spectrum of 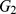 is 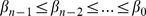. If there exists an integer 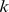
* (*
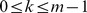
*) *such that 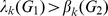, then graph 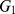 is not a subgraph of graph 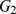.*

#### The number of walks

Paths of a graph are easier to extract and manipulate than trees and subgraphs, so GraphGrep [11] uses paths as index features. The indexes built on this kind of features are usually huge especially when graph databases are large and diverse, thus this method can be inefficient [12]. However, we find that the number of walks of length 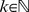 between two terminal vertices can also preserve the basic information of a graph, and the walks of a graph are much more easy to extract and manipulate than paths. Inspired by this, we extract the metrics including the number of walks with specific length as the feature for graph coding and further indexing.

Generally speaking, for each walk from vertex 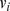 to vertex 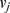 in a graph, there must exist a corresponding walk from 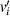 (corresponding to 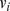) to 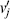 (corresponding to 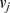) in its supergraph. Thus, we have the following lemma.

**Lemma 2** *Given two graphs 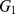 and 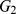, and 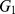 is a subgraph of 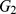. For a vertex 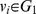, there exists a corresponding vertex 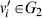, and 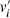 satisfies that the number of walks of length W from 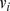 to all vertices with label l in graph 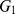 is not more than the number of walks of length W from 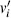 to all vertices with label l in graph 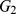.*

Applying the converse-negative proposition of Lemma 2 to vertices and graphs, we have two useful corollaries as follows.

**Corollary 5** *Given two graphs 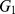 and 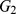, and the vertices 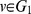 and 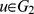 have the same vertex label. If there exists a specific vertex label l, and the vertex label l satisfies that the number of walks of length W from v to all vertices with label l in graph 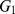 is more than the number of walks of length W from u to all vertices with label l in graph 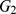, then u is not a corresponding vertex of v.*

**Corollary 6** *Given two graphs 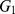 and 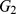, if there exists a specific vertex label l, and it satisfies that the number of walks of length W from all vertices to all vertices with label l in graph 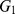 is more than the number of walks of length W from all vertices to all vertices with label l in graph 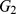, then 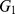 is not a subgraph of 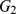.*

According to the above corollaries, we can use these features to filter out false positives. In order to speed up the comparisons between graph features, we map these features into the numerical space to generate vertex and graph codes. In the following subsection, we discuss how to generate vertex and graph codes.

### The Proposed Coding Method

In this subsection, we present the novel coding method consisting of three parts: i) *L* and 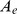 coding, ii) Laplacian spectrum coding, and iii) 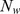 coding.

#### *L* and 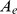 coding

For a vertex *v*, as stated in former section, *L*(*v*) is a length-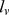 counter string to denote its vertex label, and 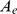
(*v*) is a length-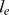 counter string to denote its adjacent edge label. For each distinct vertex (or adjacent edge) label, we use hash function to set 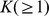 out of 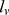 (or 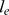) elements to 1. Then, *L*(*v*) is directly generated from the hash function of vertex label, and the code of each adjacent edge is directly generated from the hash function of adjacent edge label. By adding all adjacent edge codes with the element-wise ADD operation, we can generate 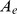
(*v*).

For a graph *G*, *L*(*G*) and 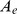
(*G*) are generated by adding *L*(*v*) and 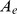
(*v*) of all vertices with the element-wise ADD operation.

**Figure 4:**
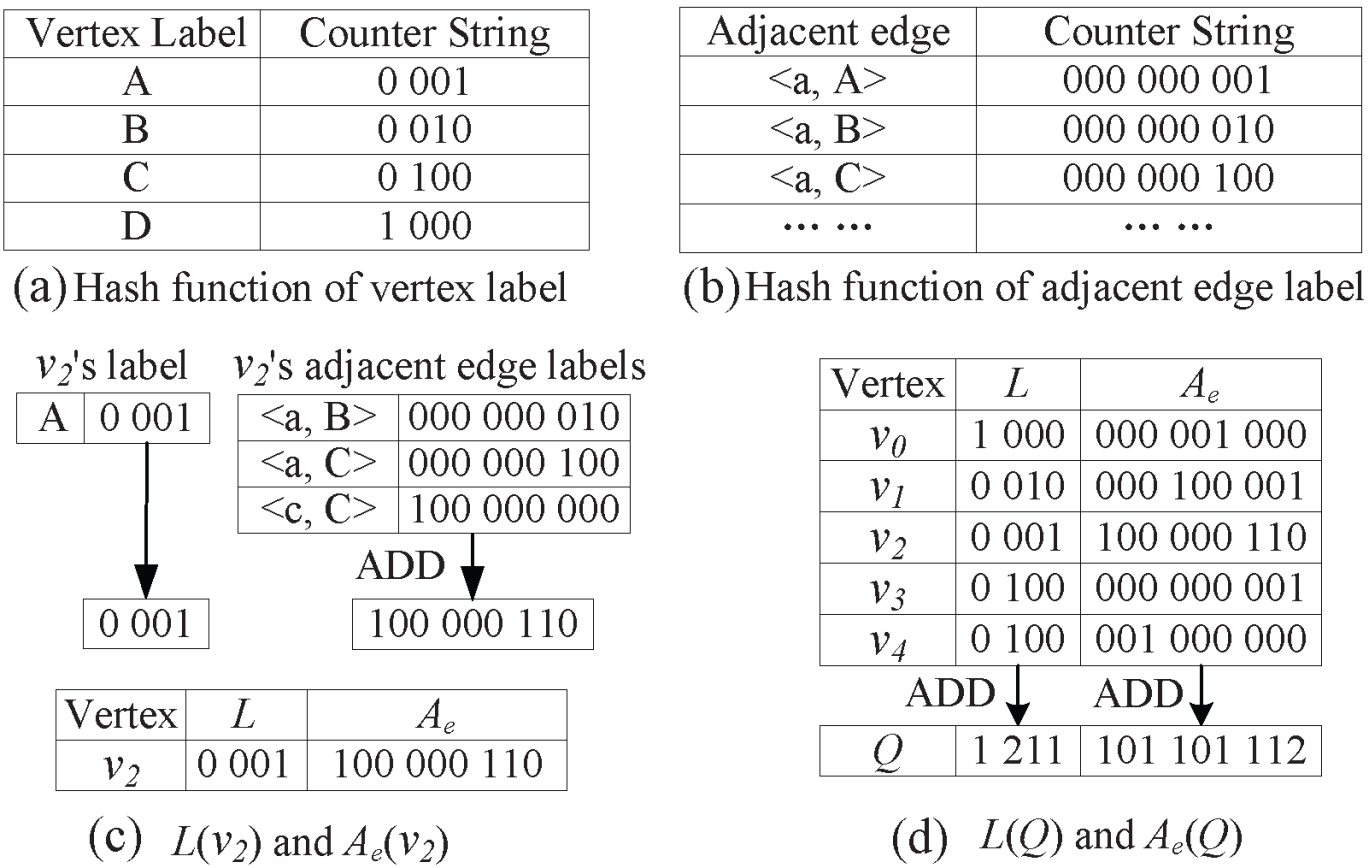
*L* and 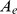 coding for graph *Q.* In this figure, (a) is the hash function of vertex label, (b) is the hash function of adjacent edge label, (c) is the generating process of 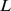 and 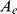 codes for vertex 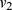, and (d) is the generating process of 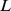 and 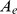 codes for graph 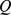.

In Fig. 4, we use vertex 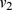 and graph *Q* as examples to illustrate the generation process of the *L* and 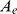 codes.

Figs. 4(a) and 4(b) are the hash functions of vertex label and adjacent edge label, respectively. For convenience sake, we denote distinct vertex (or adjacent edge) label by setting 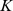 to be 1.

For vertex 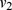, *L*(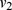) is the counter string of 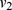 in the hash function of vertex label. In order to generate 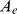
(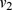), we first extract all the adjacent edges of vertex 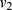: 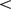
a, B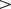, 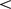
a, C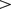 and 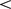
c, C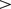. Then, we use hash function of adjacent edge label to encode each adjacent edge. Finally, we add these adjacent edge codes to generate 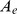
(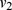), as shown in Fig. 4(c).

For graph *Q*, we combine the *L*(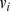) and 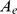
(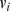) of all vertices 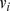 (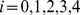) to generate its *L*(*Q*) and 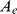
(*Q*) codes by performing the element-wise ADD operation, as shown in Fig. 4(d).

#### Laplacian spectrum coding

Suppose graph *G* has *n* vertices. For each vertex *v*, we first generate its Level-*N* Spanning Graph, and then choose some Laplacian eigenvalues of Level-*N* Spanning Graph to generate its Laplacian spectrum *Lap*(*v*). The Level-*N* Spanning Graph of a vertex is defined as follows.

**Definition 6** (Level-N Spanning Graph). *Given a graph G and a vertex 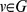,* Level*-N* Spanning Graph *of v, denoted as LNSG(G, N, v), is a subgraph representing the local structure around v, where v is a center vertex, and the vertices and edges in LNSG(G, N, v) must satisfy the follows:*

1. *for each vertex 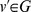, if the length of walk between v and v’ is not more than N, vertex 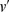 is in 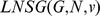;*
2. *for each edge 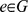, if the two terminal vertices of 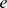 are both in LNSG(G, N, v), edge 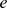 is in 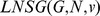.*

According to the above definition, Level-*N* Spanning Graph of a vertex is unique. By ranking the *Lap*(*v*) of all the vertices in graph *G* in non-ascending order, we obtain *Lapsseqs*(*G*).

In order to better understand the Level-*N* Spanning Graph, Table 1: Algorithm 1 lists the generation process of *LNSG*(*G*, *N*, *v*).

**Table 1:** Algorithm 1 Level-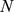 Spanning Graph Generation.

**Input**: 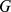 is a graph, 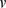 is a vertex in 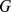 , 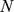 is the Level number of 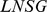 ;
**Output**: 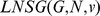 ;
1: 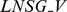 : = 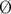 ;//The vertex set
2: 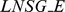 : = 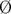 ;//The edge set
3: 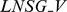 : = 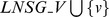 ;
4: **SEEK**( 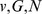 );
5: **for** each edge 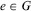 **do**
6: **if** two terminal vertices of 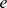 are in 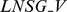 **then**
7: Insert the edge 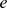 into 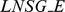 ;
8: **end if**
9: **end for**
10: **return** 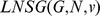 ;
**Function**: **SEEK** 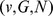
1: **if** 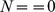 **then**
2: **return**;
3: **end if**
4: **for** each neighbor vertex 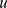 of vertex 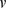 **do**
5: **if** 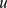 does not exist in 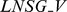 **then**
6: Insert the vertex 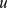 into 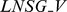 ;
7: **SEEK 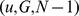 **;
8: **end if**
9: **end for**

In Table 1: Algorithm 1, Lines 1–2 initialize the vertex set and edge set of *LNSG*(*G, N, v*), respectively. Line 3 adds vertex *v* to the vertex set of *LNSG*(*G, N, v*). Line 4 uses the function *SEEK*(*v, G, N*) to find the other vertices in *LNSG*(*G, N, v*). The Function *SEEK*(*v, G, N*) uses the *depth-first-search* to traverse graph *G* and finds out all vertices in *LNSG*(*G, N, v*). Lines 5–9 look for all edges of *LNSG*(*G, N, v*). For a edge, if its two terminal vertices are both in the vertex set of *LNSG*(*G, N, v*), we add this edge to the edge set of *LNSG*(*G, N, v*).

**Figure 5:**
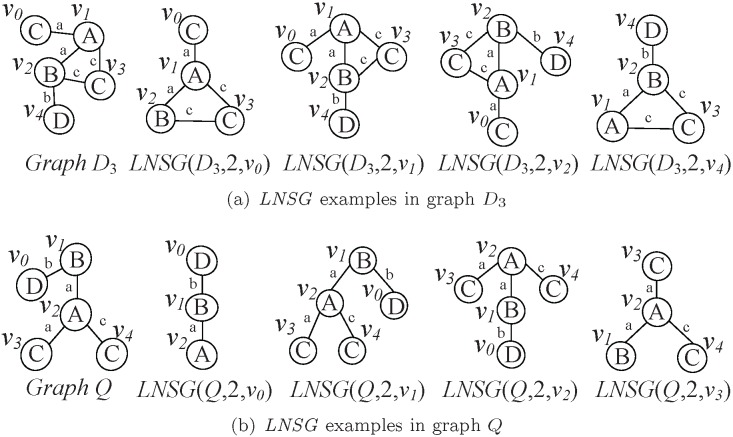
Examples of *LNSG* in graphs 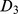 and *Q*. In the example, (a) includes the graph 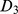 and the 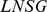 of vertices 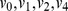; (b) includes the graph *Q* and the 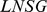 of vertices 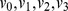.

Fig. 5 depicts the examples of Level-*N* Spanning Graph in graphs *Q* and 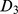, which both shown in Fig. 1. Fig. 5 (a) shows some Level-*N* Spanning Graphs for vertices 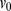, 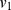, 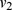 and 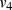 in graph 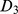. Fig. 5(b) shows some Level-*N* Spanning Graphs for vertices 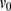, 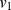, 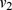 and 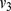 in graph *Q*. Obviously, Level-*N* Spanning Graph of 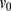, 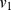, 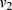 and 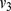 in *Q* are the subgraphs of that of 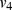, 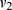, 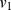 and 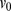 in 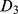, respectively.

From Fig. 5, we also find that there exists the relationship of 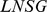 between two vertices, which are described by Lemma 3 as follows.

**Lemma 3** *Let 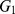 and 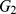 be two graphs, and 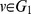 and 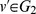 be two vertices which have the same vertex label, if 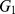 is a subgraph of 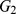 and 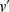 is the corresponding vertex of v in 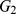, then Level-N* Spanning Graph *of vertex v is a subgraph of the Level-N* Spanning Graph *of vertex 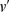.*

*Proof*. According to the subgraph isomorphism relationship, for each vertex *u* (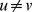) in 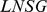
(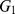, *N*, *v*), there exists a corresponding vertex 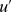 (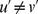) in graph 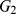. For each edge *e* in 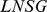
(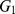, *N*, *v*), there exists a corresponding edge 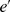 in graph 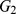. According to the definition of Level-*N* Spanning Graph, there exists a walk of length 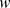 (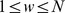) between vertices *u* and *v* in 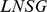
(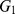, *N*, *v*). For graph 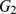, there also exists a corresponding walk of length 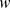 between vertices 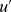 and 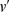. Thus, vertex 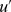 is in 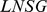
(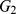, *N*, 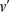). That is, the corresponding vertex of each vertex in 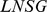
(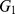, *N*, *v*) is in 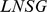
(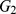, *N*, 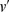). Similarly, all the corresponding edges of 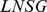
(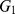, *N*, *v*) are also in 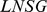
(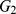, *N*, 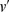). Thus, 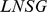
(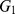, *N*, *v*) is a subgraph of 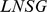
(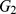, *N*, 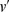).

In the proposed method, we extract some Laplacian eigenvalues of 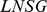
(*G, N, v*) to generate *Laps*(*v*), and generate *Lapsseqs*(*G*) via ranking the *Laps*(*v*) of all the vertices.

**Figure 6:**
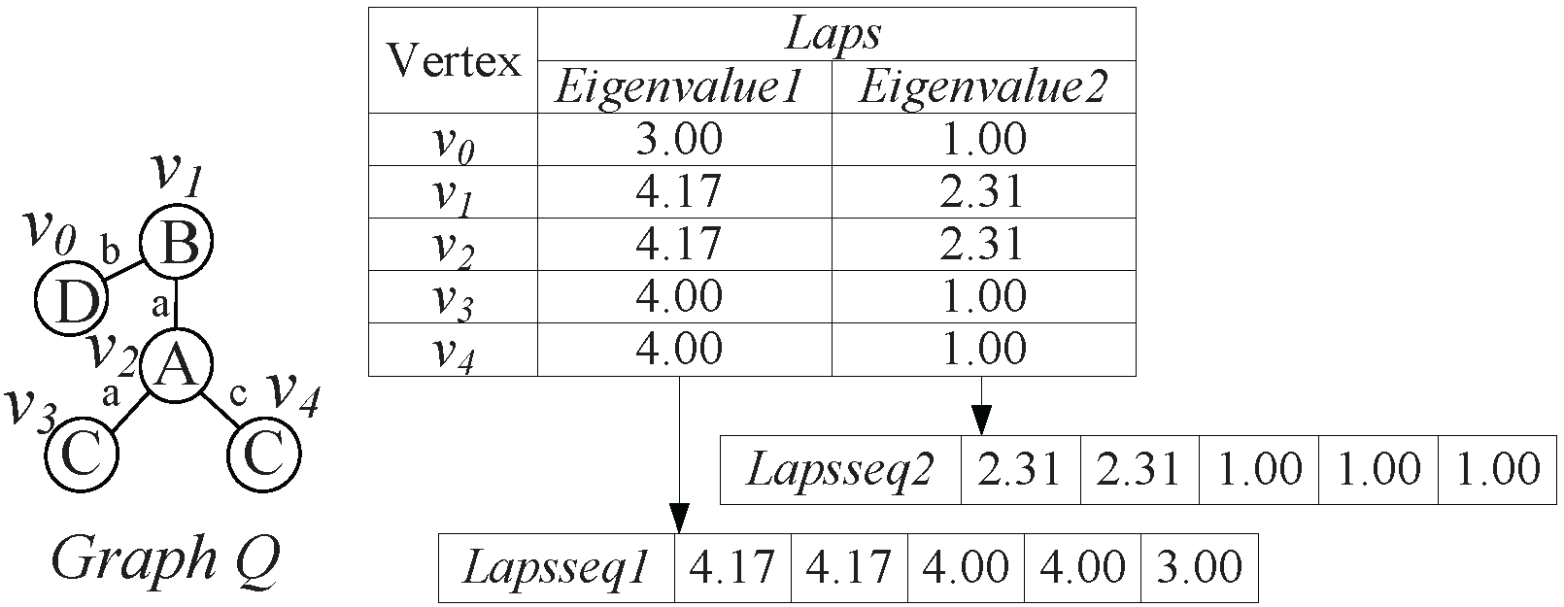
*Lap*(*v*) and *Lapsseqs*(*G*) coding for graph 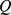. The 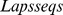 of graph 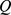 is generated by ranking the 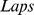 of all vertices 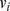 (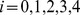).

In Fig. 6, we use graph *Q* as example to illustrate the generating process of *Laps*(*v*) and *Lapsseqs*(*G*). We first compute Laplacian spectrum of each vertex *v* in graph *Q*, and extract first two largest Laplacian eigenvalues *Eigenvalue1* and *Eigenvalue2* to generate *Laps*(*v*). According to non-ascending order, we rank the corresponding eigenvalues *Eigenvalue1* and *Eigenvalue2* of all vertices to generate *Lapsseqs*(*Q*), which contains two Laplacian spectrum sequences *Lapsseq1* and *Lapsseq2*. For convenience sake, we choose first two largest eigenvalues to denote *Laps*(*v*), and the level *N* of *LNSG* is set to 2.

#### 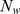 coding

A length-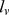 counter string is used to code 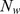
(*v*) (or 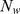
(*G*)), which is the number of walks of length 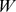. It is generated from the *W*-th power of graph *G*’s adjacency matrix. In Algebraic Graph Theory [23], there exists a lemma with respect to the number of walks of length *W* as follows.

**Lemma 4** *Let 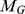 be the adjacency matrix of graph G, then the number of walks of length W from the i-th vertex of G to the j-th vertex is 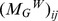 that is the entry in row i and column j of the W-th power of 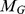.*

**Figure 7:**
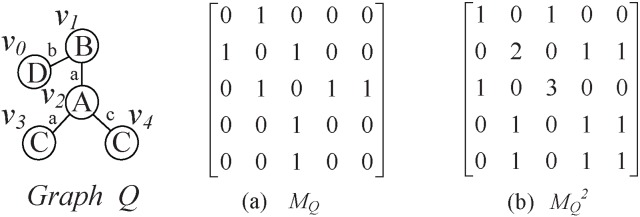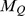 and 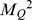. For the graph 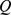, (a) is the adjacency matrix of graph 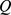; (b) is the square of the adjacency matrix of 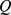.

Given graph *G* and its adjacency matrix 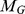, if the entry in row *i* and column *j* of 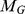 is 1, there exists a walk of length 1 between the *i*-th vertex and the *j*-th vertex in *G*. Similarly, the entry in row *i* and column *j* of the *W*-th power of adjacency matrix 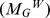 is *k* if and only if there exists 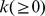 walks of length *W* between the *i*-th vertex and *j*-th vertex, where the vertices in a walk can be repetitive. Fig. 7 shows the 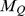 and 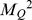 of graph *Q*, respectively.

With the *W*-th power of adjacency matrix 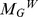 of graph *G*, for each vertex 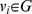, we first extract all its walks of length *W*, and generate tuple 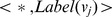 by recording the label of the terminal vertex 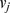 in each walk. For the distinct tuple 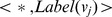, we use the hash function of walks to set 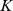 out of 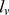 elements to 1. Then, we map each tuple 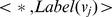 into the numerical space by using the hash function of walks, and the result is 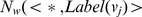). Finally, we add 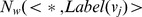) of all walks to generate 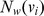 with element-wise ADD operation. Similarly, we add 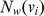 of all vertices to generate 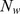
(*G*). Note that, tuple 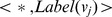 is used to represent all the walks of length *W* from vertex 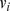 to vertex 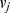, regardless the vertices or edges between them are same or not; And symbol 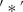 just represents the other vertices and edges appeared in a walk.

**Figure 8:**
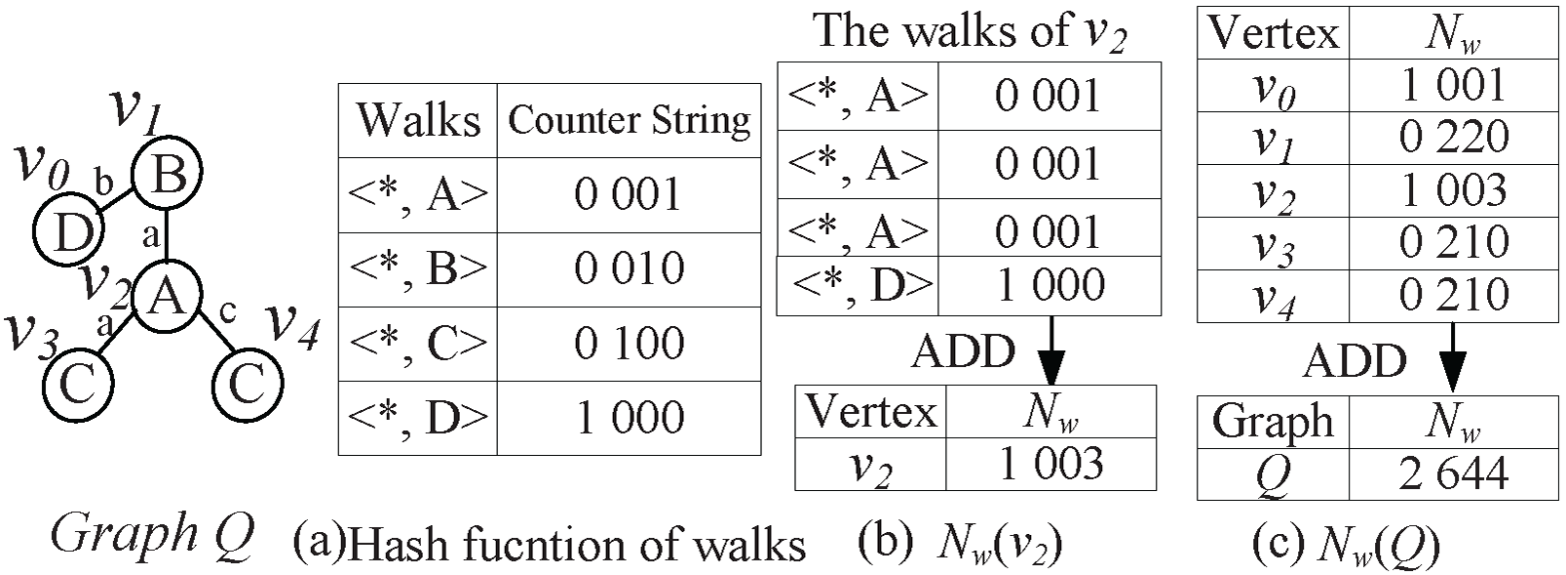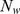 Coding. For the graph 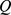, (a) is the hash function of walks; (b) is the generating process of 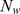 code for vertex 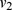; (c) is the generating process of 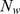 code for graph 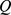.

In Fig. 8, we use vertex 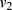 and graph *Q* as examples to illustrate the generation process of 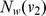 and 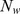
(*Q*).

Fig. 8(a) is the hash function of walks, where we represent the distinct walk by setting 1 (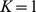) out of 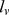 elements to 1, and the length *W* is set to 2. For vertex 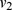, we first extract its four walks of length 2: three walks 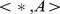 and one walk 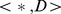 according to 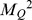 in Fig. 7, and generate 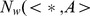) and 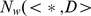) according to the hash function of walks. By adding 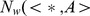) and 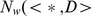) with element-wise ADD operation, we obtain 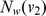, as shown in Fig. 8(b). For graph *Q*, we add 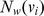 of all the vertices 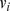 (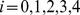) to get 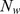
(*Q*), as shown in Fig. 8(c).

With the help of the above methods, we can extract these graph features and generate the corresponding codes. By combining *L*(*v*), 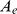
(*v*), *Laps*(*v*) and 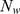
(*v*) of the vertex *v* in a graph, we can generate *vCode*(*v*, 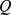), as shown in Fig. 2. By combining *L*(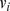), 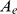
(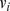), *Laps*(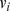) and 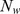
(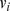) of all the vertices 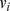 in graph *Q*, we can generate graph code *gCode*(*Q*), as shown in Fig. 3.

### Index Building

Based on the coding method, we build a graph index named LnGCode-Tree, which can improve the filtering efficiency. The construction method of the LnGCode-Tree is presented below.

LnGCode-Tree is based on the GCode-Tree, which is first proposed in GCoding [17]. Similar to S-Tree [24] and GCode-Tree, LnGCode-Tree is also used to handle the signature files, and can be efficient for reducing the number of pairwise comparisons. LnGCode-Tree is a balanced tree as well, and each index node in LnGCode-Tree has at least *m* (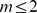) and at most *M* (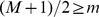) children. Different from GCode-Tree, we use the labels of vertices and adjacent edges and the number of walks to build LnGCode-Tree, while GCoding just uses the labels of vertices and adjacent edges to build GCode-Tree.

**Figure 9:**
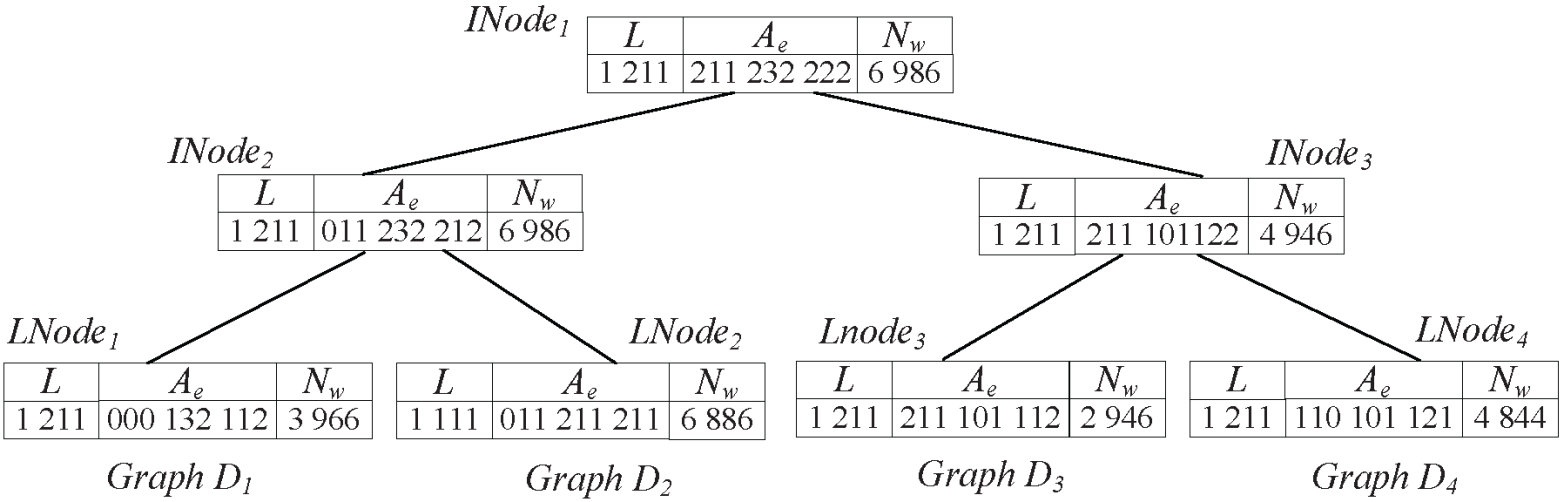
An example of LnGCode-Tree.

Fig. 9 is a LnGCode-Tree, it is built for the graphs in Fig. 1. The building process can be illustrated as follows.

For each graph 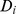, its *L*(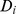), 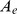
(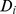) and 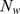
(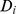) codes of 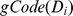 are used to build index tree. For graphs with the same *L*, 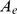 and 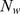 codes, a leaf node *LNode* is built. The code of *LNode* is consist of the *L*, 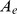 and 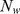 codes of graphs 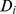 (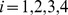), and *LNode* also contains the identities of these graphs. An intermediate node *INode* has *m* children *CNode*, its code is generated as follows: for each element *j* in *INode*, *INode*.*L*[*j*] = 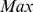
(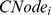.*L*[*j*]), *INode*.
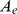
[*j*] = 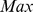
(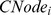.
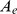
[*j*]), and *INode*.
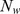
[*j*] = 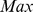
(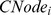.
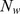
[*j*]), where 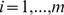.

After the index tree is built, our method generates novel two-step filtering conditions, and follows the filtering-and-verification framework to conduct query processing.

### Two-step Filtering Conditions

In this subsection, we present the two-step filtering conditions according to the properties of the graph features, and prove that these conditions satisfy the no-false-negative requirement.

#### Filtering condition of vertices

Applying Corollary 1, Lemma 3, Theorem 2 and Corollary 5 to vertices, we have a theorem as follows.

**Theorem 3** *Let 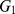 and 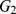 be two graphs, 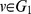 and 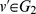 be two vertices, and 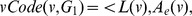

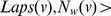
 and 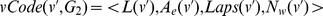 be the codes of vertices v and 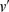 respectively. If 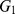 is a subgraph of 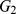 and 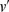 is the corresponding vertex of v, then 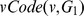 and 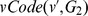 satisfy the following conditions:*

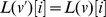
, 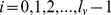;

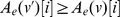
, 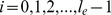;

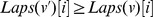
, 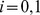;

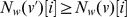
, 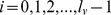.

*Proof*. Since 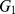 is a subgraph of 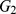, and 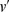 is the corresponding vertex of *v*, thus the labels of 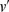 and *v* are same and their *L* codes are identical as well. That is, their *L* codes satisfy condition 1). According to Corollary 1, for each edge label *l*, the number of adjacent edges with label *l* of 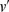 is not less than that of *v*, thus their 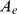 codes satisfy condition 2). According to Lemma 3 and Theorem 2, 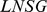
(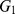, *N*, *v*) is a subgraph of 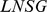
(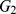, *N*, 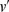), and the Laplacian spectra of 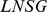
(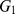, *N*, *v*) and 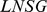
(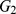, *N*, 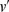) satisfy condition 3). According to Corollary 5, for each vertex label *l*, the number of walks of length *W* from *v* to all the vertices with label *l* in 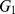 is not more than the number of walks of length *W* from 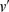 to all the vertices with label *l* in 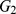, thus their 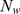 codes satisfy condition 4). Therefore, Theorem 3 is correct.

Theorem 3 shows the relationship between the codes of a vertex and its corresponding vertex. Applying the converse-negative proposition of Theorem 3 to vertices, we have the following first filtering condition.

**Filtering condition 1** *(Filtering Condition of Vertices). Let 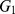 and 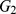 be two graphs, and 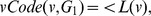

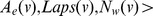
 be the code of vertex 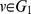, if there does not exist a vertex 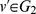, and its code 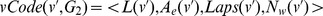 satisfies the following conditions:*

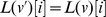
, 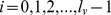;

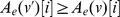
, 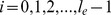;

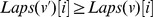
, 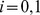;

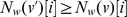
, 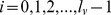.

*then 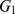 is not a subgraph of* 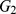.

**Lemma 5** *Filtering Condition of Vertices satisfies no-false-negative requirement for subgraph query problem.*

*Proof*. (Proof by contradiction) We assume the *Filtering Condition of Vertices* does not satisfy the no-false-negative requirement. Let 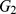 be a graph and 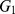 be its subgraph, and *Filtering Condition of Vertices* do not satisfy the no-false-negative requirement if and only if 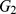 can be pruned by *Filtering Condition of Vertices*. That is, for a specific vertex 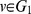, there does not exist a vertex 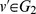, and the 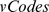 of 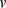 and 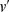 satisfy the conditions 1), 2), 3) and 4) in *Filtering Condition of Vertices*. According to Theorem 3, for each vertex 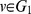, there must exists a corresponding vertex 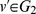, and the 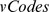 of 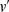 and *v* satisfy the conditions 1), 2), 3) and 4) in *Filtering Condition of Vertices*. Thus, graph 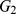 cannot be pruned by *Filtering Condition of Vertices*. This contradicts the assumption. Therefore, Lemma 5 is correct.

#### Filtering conditions of graphs

Applying Corollary 2, Lemma 3, Theorem 2 and Corollary 6 to graphs, we have another theorem as follows.

**Theorem 4** *Let m and n (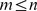) be the numbers of vertices and 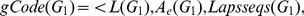

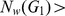
 and 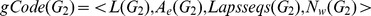 be the codes of graphs 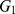 and 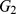 respectively, if 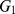 is a subgraph of 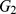, then their graph codes 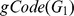 and 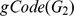 satisfy the following conditions:*

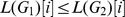
, 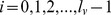;

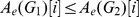
, 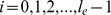;

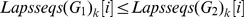
, 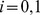 and 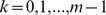;

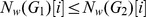
, 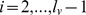.

*Proof*. (sketch) The conditions 1), 2) and 4) can be directly derived from Corollary 2 and Corollary 6. Condition 3) is proved as follows. Since 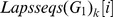 is a sorted list in non-ascending order, there exist 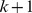 vertices 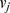 (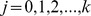) in 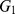, and the Laplacian eigenvalue 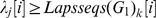. According to Theorem 2, for each vertex 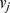 (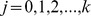) in 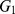, there exists a corresponding vertex 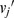 in 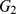, and the Laplacian eigenvalues 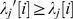 (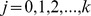). That is, 
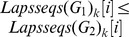
 (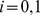). Thus the condition (3) is correct.

Applying the converse-negative proposition of Theorem 4 to graphs, we have the second filtering condition.

**Filtering condition 2** *(Filtering Condition of Graphs). Let m and n (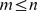) be the numbers of vertices and 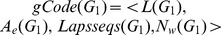 and 
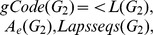

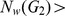
 be the codes of graphs 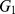 and 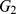 respectively, if 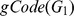 and 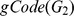 do not satisfy the following conditions:*

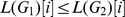
, 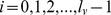;

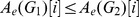
, 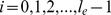;

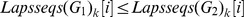
, 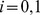 and 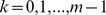;

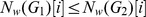
, 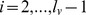.

*then 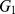 is not a subgraph of* 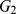.

**Lemma 6** *Filtering Condition of Graphs satisfies the no-false-negative requirement for subgraph query problem.*

*Proof*. Similar to Lemma 5, this lemma can be proved by contradiction according to Theorem 4.

### Filtering and Verification

Based on the index and filtering conditions, we follow the filtering-and-verification framework to query subgraphs.

Firstly, we use two-step filtering conditions to filter out false positives. In the first step, we traverse the LnGCode-Tree of graph database with *Filtering Condition of Graphs*. Specifically, the graph code 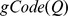 of query graph *Q* is compared with the intermediate node 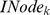. If there exists an element *i*, and it satisfies one of these conditions: i) 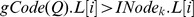; ii) 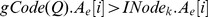; or iii) 
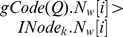
, then the children of 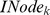 are pruned; otherwise, the graph code 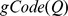 is compared with each child of 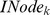. For the leaf node 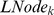, if there exists an element *i*, and it satisfies one of these conditions: i) 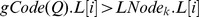; ii) 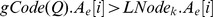; or iii) 
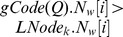
, then the graphs contained in 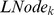 can be pruned as false positives; otherwise, the graphs contained in 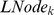 are added to candidate graphs. After traversing LnGCode-Tree, LnGCoding filters out some false positives, so the graph database is reduced. Then we compare the *Lapsseqs* of the query graph with those of the reduced graph database, since LnGCode-Tree only includes *L* and 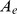, 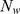 codes. Through this step, we obtain the primary candidate graph set for the query graph.

This step can be illustrated by the graphs in Fig. 1 and the corresponding LnGCode-Tree in Fig. 9. When traversing 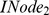, we find that 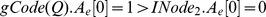, thus graphs 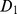 and 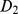 are pruned. When traversing 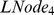, we find that 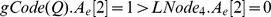, so graph 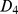 is pruned. Then, by comparing the *Lapsseqs* of query graph *Q* and graph 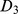, we find 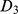 is a candidate of *Q*.

In the second step, we use *Filtering Condition of Vertices* to filter out more false positives. Specifically, we compare each vertex code of the query graph with all the vertex codes of each graph in the primary candidate graph set until all the candidate vertices of this vertex have been found. By now, the candidate graph set and the candidate vertex set are generated.

**Figure 10:**
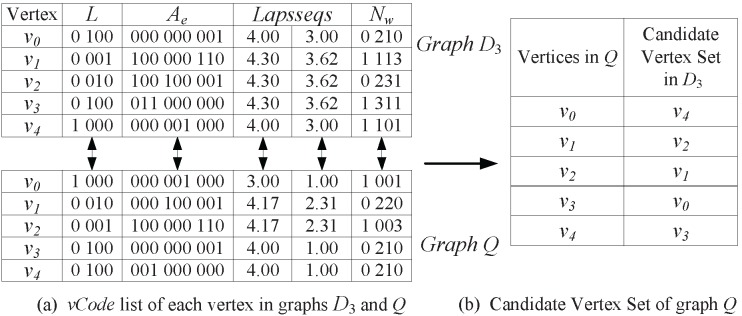
A Filtering Example. For the labeled graphs 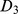 and 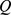, (a) lists the 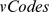 of all vertices in 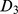 and 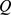; (b) lists the candidate vertices set for each vertex in 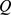.

In Fig. 10, we use graph *Q* as query graph and 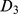 as the primary candidate graph set to illustrate the second step filtering process.

The vertex codes of all vertices in graphs 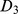 and *Q* are shown in Fig. 10(a). After filtering with *Filtering Condition of Vertices*, we generate the candidate vertex set of each vertex in query graph *Q*, as shown in Fig. 10(b). For each vertex in *Q*, there exist the corresponding candidate vertices in 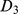. Thus, 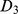 is a candidate graph of *Q*.

After the filtering is finished, in the verification phase, we use the state-of-the-art subgraph isomorphism algorithm VF2 [25], [26] to validate each candidate graph, and obtain the supergraph set for a query graph.

## Experimental Results and Discussion

In this section, after introducing the data source, the benchmark methods and parameter setting, and the evaluation criteria, we report the experimental results on efficiency comparison of the different methods, and test the scalability of our method.

### Data Source

In this study, both real and synthetic graph databases are used.

#### Real graph database

The AIDS antiviral screen database contains 43,905 classified chemical molecules, and is publicly available. Many researchers such as Yan et al. [12], Shang et al. [16], Zou et al. [17], and He and Singh [18] used one of its subset to test their methods, we chose it as benchmark data as well.

The subset consists of 10,000 graphs as default database. On average, each graph has 25.4 vertices and 27.3 edges, which means that most of graphs in this real graph database are sparse graphs. Six query graph sets *Q*4, *Q*8, *Q*12, *Q*16, *Q*20 and *Q*24 are used to validate the efficiency of subgraph querying methods. Each query graph set 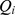 (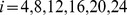) consists of 1,000 query graphs with *i* edges.

#### Synthetic graph database

GraphGen [27] is a synthetic graph generator. In order to test the performance of existing methods on dense graphs, Han et al. [19], [20] used it to generate the synthetic graph database Synthetic.10K.E30.D5.L50. The cardinality of the synthetic database is 10,000, the average size of graphs is 30, the density for each graph is 0.5, and the number of vertex/edge labels is 50.

### Benchmark Methods and Parameter Setting

#### Benchmark methods

The representative methods gIndex [12], FG-Index [13], Tree+delta [15], SwiftIndex [16], GCoding [17], and Closure-tree [18] are selected to be compared with our method. Since LsGCoding [21] aims at coding graphs with unlabeled edge, and optimizes the subgraph isomorphism algorithm according to the properties of graphs with unlabeled edge, thus in our experiments on graph databases with labeled edges, we do not compare LsGCoding with our method.

All these methods are implemented on the iGraph framework [19], [20], this enables fair performance comparisons for different methods.

#### Parameter setting

**Figure 11:**
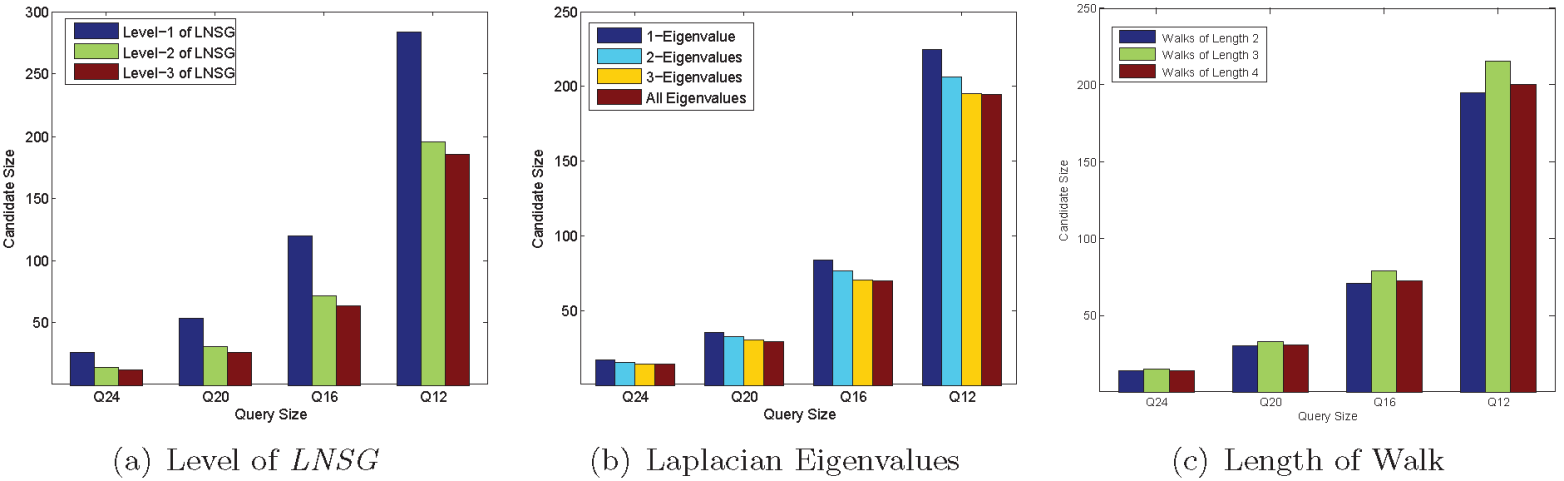
Impacts of Parameters on Candidate Set Size. (a) Level of 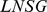; (b) Laplacian eigenvalues; (c) Length of Walk.

Our proposed method has three parameters: the level of 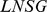, the number of first largest Laplacian eigenvalues, and the length of walks. Fig. 11 shows the impact of these parameters on the real graph database.

Fig. 11(a) shows the impact of the level of 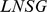 on the candidate set size. It indicates that when we choose more levels of 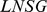, the candidate set size will become smaller. However, the more levels of 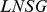 we choose, the more time will be consumed in computing Laplacian spectrum. Moreover, choosing 3 or more levels cannot lead to significant reduction in the candidate set size. Therefore, the level *N* of 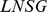 is set to 2.

Fig. 11(b) shows the impact of Laplacian eigenvalues on the candidate set size. We observe that choosing more Laplacian eigenvalues can reduce the size of the candidate graph set, but will result in the larger graph code database and more code comparison time. At the same time, choosing 4 or more Laplacian eigenvalues cannot lead to significant reduction in the candidates set size. Therefore, we choose the first three largest eigenvalues in our method.

Fig. 11(c) shows the impact of the length of walks on the candidate set size. From it we know that longer length of walks will result in more computation time of matrix 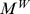, and choosing 3 or greater length cannot lead to significant reduction in the candidate set size. Thus we set the length *W* to 2.

As recommended in [17] and [28], the length of *L*, 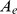 and 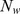 codes are set to 30 (i.e. 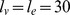).

For methods gIndex, FG-Index, Tree+delta, SwiftIndex, GCoding and Closure-tree, the recommended parameter values are used. That is, for all substructures based index methods, the support threshold is set to 10%, and the maximum feature size *maxL* is set to 10. For gIndex and SwiftIndex, 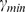 is set to 2. For FG-Index, 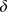 is set to 0.1. For gIndex, the same size-increasing function as in [12] is followed. For GCoding, the level *N* of 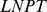 is set to 2 and the number of eigenvalues to 2.

### Evaluation Criteria

A subgraph query algorithm usually consists of two processes: i) coding and indexing, and ii) subgraph querying. In this section, we briefly introduce some criteria metrics used to evaluate the efficiency of these two parts.

#### Criteria for coding and indexing

The coding and indexing time and the index size for both graph codes and the index tree are used in this process.

- Coding and Index Time. The coding and indexing time is the run time used to encode both graphs and their vertices and build the index tree. A less coding and indexing time means higher performance in this process.
- Index Size. The index size is the size of space used to store both the graph codes and the index tree. In the filtering phase, much time is spent on accessing a larger index, so it partly impacts the filtering efficiency.

#### Criteria for subgraph querying

The candidate set size, the filtering time, the verification time and the response time are used in this process.

- Candidate Set Size. The candidate set size is the number of candidate graphs for each query graph. For each subgraph query algorithm, a smaller candidate set size implies higher filtering efficiency.
- Filtering Time. For each subgraph query method, the Filtering time is the run time to traverse the index to filter out false positives and generate the candidate set. A less filtering time implies higher filtering efficiency.
- Verification Time. For each subgraph query method, the verification time is the run time to verify each candidate and generate the result set. A less verification time implies higher verification efficiency.
- Response Time. For each subgraph query method, the response time is defined as the sum of the filtering time and the verification time. A less response time means the higher of querying efficiency.

Our experiments evaluate the efficiency of different subgraph query methods. For each subgraph query method, the run time is the most important criterion in each phase. Thus, in the first phase, the coding and index time is the primary criterion; and in the second phase, the response time is the primary criterion.

### Performance on Real Graph Database

#### Performance of coding and indexing

**Figure 12:**
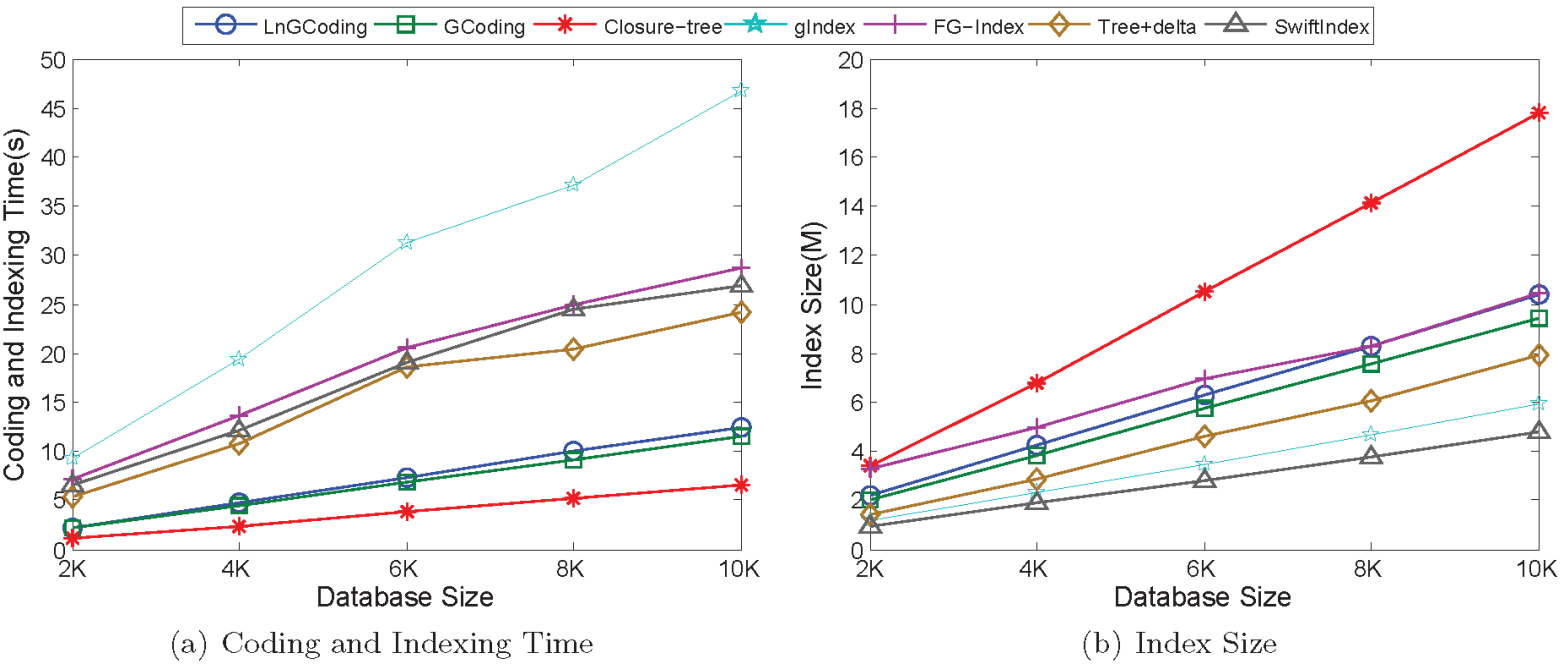
Performance of Coding and Indexing on Real Data. (a) Coding and Indexing Time; (b) Index Size.

Fig. 12 shows the performance of the seven methods on the real graphs in the coding and indexing process.

*Coding and Indexing Time*. Fig. 12(a) shows the coding and indexing time of all the seven methods on the real graph database. From it we observe that, with the increasing of database size from 2 K to 10 K, the coding and indexing time of each methods is increasing.

Compared with Closure-tree, since LnGCoding must compute the expensive Laplacian spectrum, thus the coding and indexing time in LnGCoding is more than that of Closure-tree.

In the coding based index methods, LnGCoding computes not only the Laplacian spectrum but also the number of walks. Thus, the coding and indexing time in LnGCoding is the larger than that of GCoding.

For the substructure based index methods, they extract graph features via expensive frequent subgraph or subtree mining. Thus, their coding and indexing time is greater than that of LnGCoding.

In a word, the coding and indexing time of our method is much less than that of the substructure based index methods, and is comparable with those of GCoding and Closure-Tree.

*Index Size.* Fig. 12(b) shows the index sizes of the seven methods on the real graph database. From it we know that, when the database size is increasing from 2 K to 10 K, the index size of each method is also increasing.

The index size of Closure-tree is more than that of LnGCoding, since the coding based index methods both map the information of graph features into the numerical spaces, which can save the store space.

The index size of LnGCoding is more than that of GCoding, since the code in LnGCoding consists three parts: the labels of vertices and adjacent edges, the Laplacian spectrum, and the number of walks; while the code in GCoding contains two parts: the labels of vertices and adjacent edges, and the graph spectrum.

Since FG-Index generates all frequent subgraphs and all infrequent edges for completeness, its index size is greater than that of LnGCoding. For the other substructure based index methods, their index sizes are less than that of LnGCoding, because the sizes of mined features or the numbers of mined features are small [19].

#### Performance of querying

**Figure 13:**
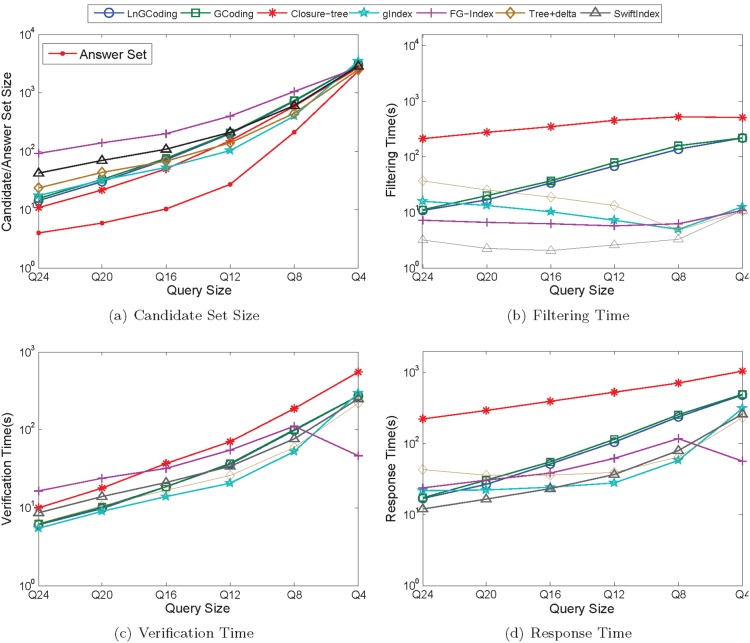
Performance of Querying on Real Data. (a) Candidate Set Size; (b) Filtering Time; (c) Verification Time; (d) Responde Time.

Fig. 13 shows the performance of the seven methods on the real graphs in querying process.

*Candidate Set Size*. Fig. 13(a) shows that, when query graph set is varying from *Q*24 to *Q*4, the candidate set size of each method is increasing. This is because the answer set is increasing. When query size is larger, such as *Q*24 and *Q*20, the candidate set sizes of the clustering based and coding based index methods are less than those of the substructure based index methods; while when the query size is smaller, such as *Q*8 and *Q*4, the candidate set sizes of the clustering based and coding based index methods are greater than those of the most substructure based index methods. The reason is that for these substructure based index methods, more features are mined on the smaller sized graphs than on the larger sized graphs.

Closure-tree prunes more false positives than that of LnGCoding, since it conducts the pseudo subgraph isomorphism testing, which is similar to the exact subgraph isomorphism algorithm.

Different from the graph spectrum in GCoding, LnGCoding uses Laplacian spectrum and the number of walks as graph features, thus the candidate set size of LnGCoding is less than that of GCoding.

For the substructure based index methods, since their mined index features are less for larger sized query graphs than for smaller sized query graphs, their candidate set sizes are greater than those of LnGCoding when the query graph sets are *Q*24 and *Q*20. When the size of the query graph is smaller, such as *Q*12, *Q*8 and *Q*4, the candidate set sizes of gIndex, Tree+delta and SwiftIndex are less than those of LnGCoding. FG-Index generates the largest candidate set size, this is because it traverses the index to find a subset of mined features which is a subgraph of the query graph. This means it does not find out all subgraphs of a query graph from its index.

*Filtering Time*. Fig. 13(b) shows that, when the query graph set is varying from *Q*24 to *Q*4, the filtering time of the clustering based and coding based index methods is increasing, while the filtering time of the substructure based index methods is decreasing. The reason is that for the substructure based index methods, there are less index features in query graph set *Q*4 than in *Q*24, thus there are less comparisons between the query graph and the index features in *Q*4 than in *Q*24.

From Fig. 13(b) we also know that the filtering time of Closure-tree is the largest, as it conducts the pseudo subgraph isomorphism testing that is quite time consuming.

The vertex and graph codes of LnGCoding are more complex than those of GCoding, and the code comparison of the former is more expensive than that of the latter. Thus, the filtering time of LnGCoding is slightly greater than that of GCoding.

For the substructure based index methods, since their index sizes are less than that of LnGCoding, they traverse the index to filter out false positives with less time. Thus, the filtering time of most of them is less than that of LnGCoding.

*Verification Time*. Fig. 13(c) shows that, when the query graph set is varying from *Q*24 to *Q*4, the verification time of most methods are increasing.

Under the iGraph framework, Closure-tree employs a java bytecode analyzer to verify candidates, while LnGCoding uses the-state-of-art subgraph isomorphism algorithm VF2 [25] to verify candidates. Although Closure-tree has the smaller candidate set size than that of LnGCoding, the verification time of Closure-tree is more than that of LnGCoding.

For the graph coding based index methods, the candidate set size of LnGCoding is slightly less than that of GCoding, so the verification time of the former is also slightly less than that of the latter.

For the substructure based index method FG-Index, its verification time is less than that of LnGCoding for query graph set *Q*4, and is more than those of LnGCoding for other query graph sets. The reason is that FG-Index employs a verification free strategy: when the query graph is an indexed feature, it directly reports the answer set without verification. Since *Q*4 has most indexed features for all query graph sets, the verification time of FG-Index is less than those of the other methods.

The verification time of gIndex is slightly less than those of LnGCoding for query graph sets *Q*24 and *Q*20. The reason lies in that, the candidate set sizes of gIndex are slightly more than those of LnGCoding, and the index size of gIndex is much less than that of LnGCoding, so its cost for finding the candidate graphs is less than that of LnGCoding. For other query graph sets, the verification time of gIndex is less than those of LnGCoding, since the candidate set sizes of gIndex are much less than those of LnGCoding on these query graph sets.

The verification time of LnGCoding is less than those of Tree+delta for query graph sets *Q*24 and *Q*20, and is greater than those of Tree+delta for query graph sets *Q*16, *Q*12, *Q*8 and *Q*4. It is because the candidate set sizes of the former are much less than those of the latter for query graph sets *Q*24 and *Q*20, and the candidate set sizes of the former are greater than those of the latter for query graph sets *Q*16, *Q*12, *Q*8 and *Q*4.

Due to the sizes of candidate set, the verification time of LnGCoding is less than those of SwiftIndex for query graph sets *Q*24, *Q*20 and *Q*16, and is greater than those of SwiftIndex for query graph sets *Q*8 and *Q*4. For query graph set *Q*12, the verification time of LnGCoding is slightly more than that of SwiftIndex, since the candidate set size of SwiftIndex is slightly more that of LnGCoding for query graph set *Q*12, and the index size of SwiftIndex is much less than that of LnGCoding.

*Response Time*. Fig. 13(d) shows that, when the query graph set is varying from *Q*24 to *Q*4, the response times of most methods are increasing.

The filtering time and the verification time of Closure-tree both are the largest, so its response time is the biggest.

Since the filtering time of LnGCoding is much less than that of GCoding, and its verification time is smaller than or comparable to that of the latter, the response time of LnGCoding is less than that of GCoding. This means that LnGCoding performs best on the real graph database among the clustering based and coding based index methods.

For the substructure based index method SwiftIndex, its filtering time is much less than those of LnGCoding on all query graph sets, so the response time is less than those of the latter as well.

For the query graph set *Q*24, the filtering time of LnGCoding is much less than that of gIndex, thus its response time is less than that of the latter. For other query graph sets, the filtering time and verification time of LnGCoding both are greater than those of gIndex, so its response time is greater than those of the latter.

For the query graph sets *Q*24 and *Q*20, the filtering time of LnGCoding is much less than those of Tree+delta, and the verification time of LnGCoding is much less than those of FG-Index, thus its response time is less than those of Tree+delta and FG-Index. For other query graph sets, the filtering time of LnGCoding are greater than or much greater than those of Tree+delta and FG-Index, thus its response time is greater than those of Tree+delta and FG-Index.

According to the experimental results on real data, our method works well with larger query size. For the small query size, our method is faster than GCoding and Closure-Tree, but slower than the substructure based index methods.

In a word, for the real data experiment, the response time of LnGCoding is not as good as substructure-based methods like SwiftIndex, but LnGCoding outperforms these substructure-based methods regarding coding and indexing.

### Performance on Synthetic Graphs

#### Performance of coding and indexing

**Figure 14:**
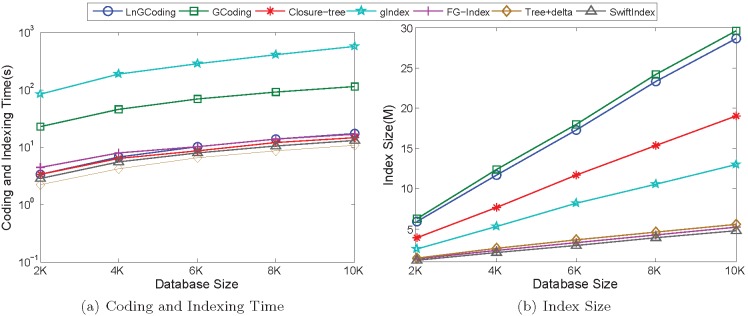
Performance of Coding and Indexing on Synthetic Data. (a) Coding and Indexing Time; (b) Index Size.

Fig. 14 shows the performance of the seven methods on the synthetic graphs in the coding and indexing process.

*Coding and Indexing Time*. Fig. 14(a) shows the coding and indexing time of the seven methods on the synthetic graph database. From it we know that, with the increase of the database size, the coding and indexing time of each method is also increasing.

Since LnGCoding must compute the expensive graph spectrum, thus the coding and indexing time of LnGCoding is greater than that of Closure-tree.

**Figure 15:**
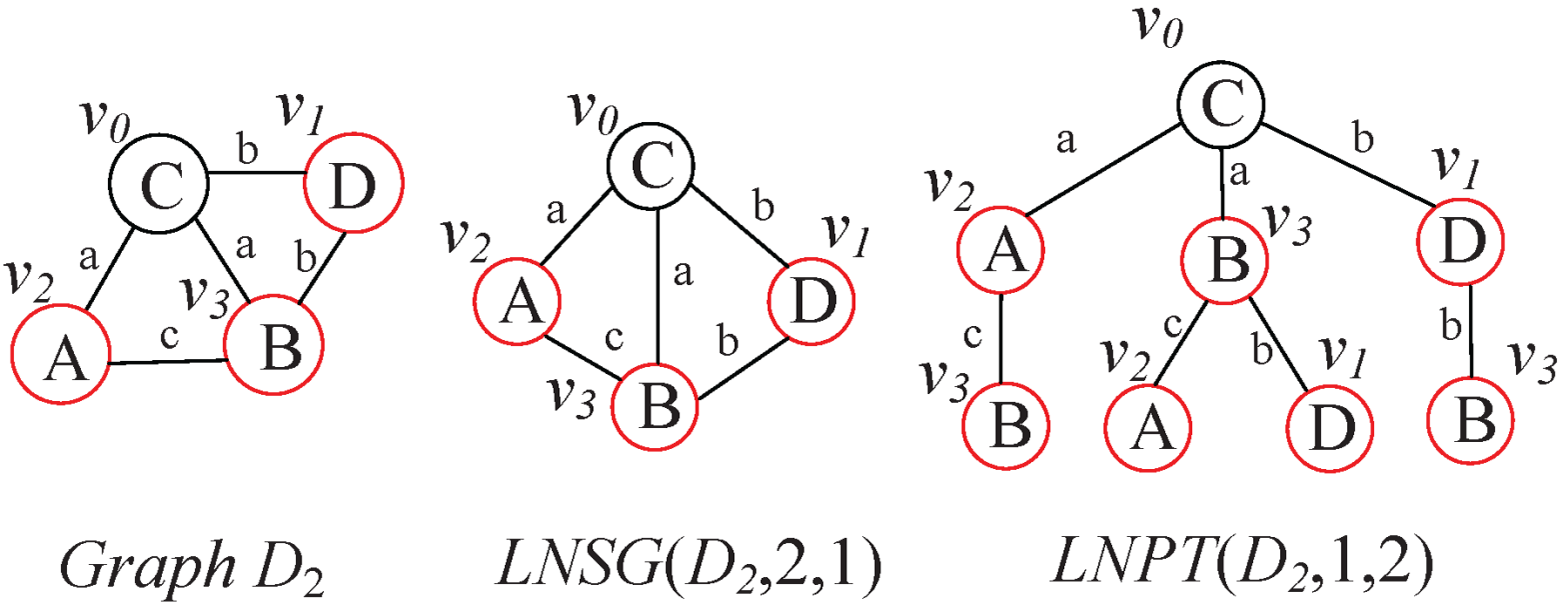
*LNSG* and *LNPT* of 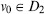.

When computing graph spectrum, GCoding generates Level-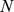 Path Tree (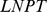) and LnGCoding generates 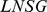. However, 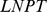 is built by adding reduplicate vertices, and 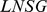 is generated without any reduplicate vertices. Fig. 15 shows the differences between *LNSG* and 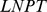 of vertex 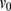 in graph 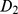, which occurred in Fig. 1.

From Fig. 15 we observe that 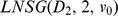 contains 4 vertices, but 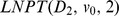 contains 8 vertices. Obviously, 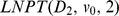 contains four reduplicated red vertices: one vertex 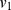, one vertex 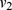 and two vertices 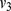. Since the computational complexity of graph spectrum is 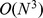 (*N* is the number of vertices), GCoding is much more time consuming than LnGCoding, specially when the graph is dense. In the synthetic graph database, most graphs are dense. Thus, the coding and indexing time of LnGCoding is less than that of GCoding. Meanwhile, we can see that 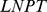 does not contain the cycles occurred in the graph, which degrades the filtering efficiency.

For the substructure based index methods, the coding and indexing time of gIndex is the largest due to it mines much more features, and the coding and indexing time of Tree+delta and SwiftIndex is smaller than that of LnGCoding because the mined features are less.

In a word, the coding and index time of our method is much less than that of gIndex and GCoding, and is comparable with the fastest method Tree+delta.

*Index Size*. Fig. 14(b) shows the index size of the seven methods on the synthetic graph database. From it we know that, with the increase of database size, the index size of each method is also increasing.

Since most of synthetic graphs are dense, LnGCoding must use more space to store the Laplacian spectrum. Thus, the index size of LnGCoding is greater than that of Closure-tree.

For the coding based index methods, GCoding generates 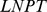 by adding some reduplicate vertices while LnGCoding generates 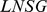 without any reduplicate vertices, thus the index size of LnGCoding is smaller than that of GCoding.

For the substructure based index methods, the mined features of gIndex are much more than those of others, so its index size is greater as well. Moreover, the mined index features of these substructure based index methods are smaller subgraph or substructures, thus the index size of LnGCoding is bigger than those of these methods.

#### Performance of querying

**Figure 16:**
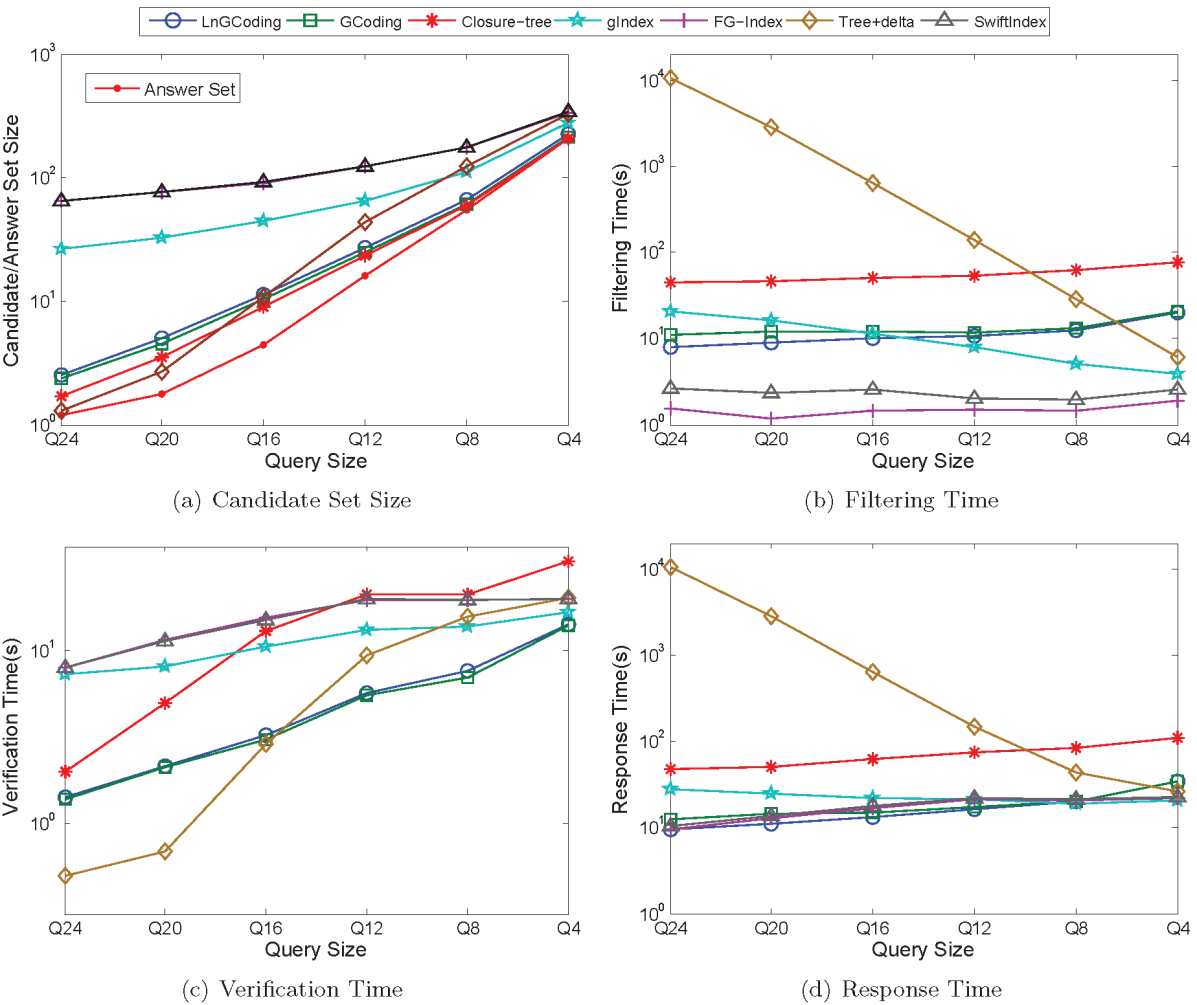
Performance of Query Processing on Synthetic Data. (a) Candidate Set Size; (b) Filtering Time; (c) Verification Time; (d) Responde Time.

Fig. 16 shows the performance of the seven methods on the synthetic graphs in querying process.

*Candidate Set Size*. Fig. 16(a) shows the candidate set sizes of the seven methods on the synthetic graph database. We observe that, when the query graph size is varying from *Q*24 to *Q*4, the candidate set size of each method is increasing, this is because the answer set size of each method is increasing.

Closure-tree conducts the pseudo subgraph isomorphism testing in the filtering phase, thus its candidate set size is less than that of LnGCoding.

For the coding based index methods, GCoding and LnGCoding roughly have the same number of candidates.

For the substructure based index methods, the candidate set sizes of Tree+delta are less than those of LnGCoding on query graph sets *Q*24, *Q*20 and *Q*16, since it takes too much time to filter out false positives on these query graphs. For other query graph sets, the candidate set sizes of LnGCoding are smaller than those of Tree+delta. For the other substructure based index methods, as their index features are not effective for dense graphs, their candidate set sizes are greater than those of LnGCoding.

*Filtering Time*. Fig. 16(b) shows the filtering time of the seven methods on the synthetic graph database.

Since Closure-tree conducts the pseudo subgraph isomorphism testing to filter out false positives, thus its filtering time is much greater than that of LnGCoding.

For the coding based index methods, GCoding filters out more false positives than that of LnGCoding, thus its filtering time is greater than that of LnGCoding.

For the substructure based index methods, gIndex has the most mined features, and the sizes of most index features are small. For the query graph sets *Q*24, *Q*20 and *Q*16, gIndex uses ineffective features to minimize the number of candidates, thus its filtering time is greater than those of LnGCoding on these query graph sets. For other query graph sets, its filtering time is less than those of LnGCoding.

The filtering time of Tree+delta is also greater than that of LnGCoding except for *Q*4. This is because that the query graphs contain many cycles in dense graph database, and Tree+delta mines too many graph features to its “delta”, which is very time consuming.

The mined features of FG-Index and SwiftIndex are not effective for dense graph database, they filter out much less false positives than LnGCoding. Thus, their filtering time are less than that of LnGCoding for all query graph sets.

*Verification Time*. Fig. 16(c) shows the verification time of the seven methods on the synthetic graph database. From it we know that, with the decrease of the query graph size, the verification time of each method is also increasing. This is because the candidate set size of each method is increasing.

Since Closure-tree follows iGraph’s original implementation exactly using a java bytecode analyzer, thus its verification time is greater than that of LnGCoding.

For the coding based index methods, the candidate set size of GCoding is slightly less than that of LnGCoding, so its verification time is slightly smaller than that of LnGCoding.

For the substructure based index method Tree+delta, its candidate set sizes are less than those of LnGCoding for query graph sets *Q*24, *Q*20 and *Q*16, so its verification time is smaller than those of LnGCoding on these query graph sets. As for the other query graph sets, since the candidate set sizes of Tree+delta are greater than those of LnGCoding, its verification time is also greater than those of LnGCoding.

For the other substructure based index methods, their candidate set sizes are much more than those of LnGCoding, thus their verification time is also greater than those of LnGCoding. Note that, the verification time of FG-Index is not the least for query graph set *Q*4, since there are not many frequent features on query graph set *Q*4.

*Response Time*. Fig. 16(d) shows the response time of the seven methods on the synthetic graph database.

Since Closure-tree has the more filtering time and verification time than those of LnGCoding, thus its response time is bigger than that of LnGCoding.

For the coding based index methods, the filtering time of LnGCoding is much less than that of GCoding, thus its response time is less than that of GCoding.

The substructure based index method Tree+delta takes much more time to filter out false positives, thus its response time is greater than that of LnGCoding except for *Q*4.

For the other substructure based index methods, their filtering time is much less than that of LnGCoding for *Q*4, thus their response time is less than that of LnGCoding on query graph set *Q*4. As for the other query graph sets, these methods’ verification time is much greater than those of LnGCoding, thus their response time is greater than those of LnGCoding. Thus, the response time of LnGCoding is the least among all methods except for query graph set *Q*4, and our method performs best on dense graph database.

In a word, for the synthetic data with dense graphs, LnGCoding has the best response time and similar coding and indexing time as the fastest methods; FG-Index and SwiftIndex are close competitors to LnGCoding regarding both evaluation measures.

From the experiments over both real and synthetic graph data, we can find that, although none of these methods outperforms others on all the databases, our proposed method does outperform competitors when graphs are dense.

### Scalability Test

In order to evaluate the scalability of LnGCoding, we conduct experiments on the synthetic graph data with different sizes and distinct vertex labels.

The synthetic graph data consists of the ten graph databases that are generated with a graph generator, which is developed by Kuramochi and Karypis [29] and also used in [18] and [17], by varying the cardinality and the vertex labels. Three subsets are selected as the query graph sets to test the scalability of our method.

#### Performance on graphs with varying sizes

In this experiment, we generated five databases *D5K*, *D10K*, *D20K*, *D30K* and *D40K* by varying the database cardinality. For database *DnK* (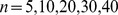), *nK* (i.e. 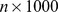) graphs are included. The query graph sets are *Q*10, *Q*15 and *Q*20, where each query graph set *Qi* consists of 1,000 query graphs with *i* edges.

**Figure 17:**
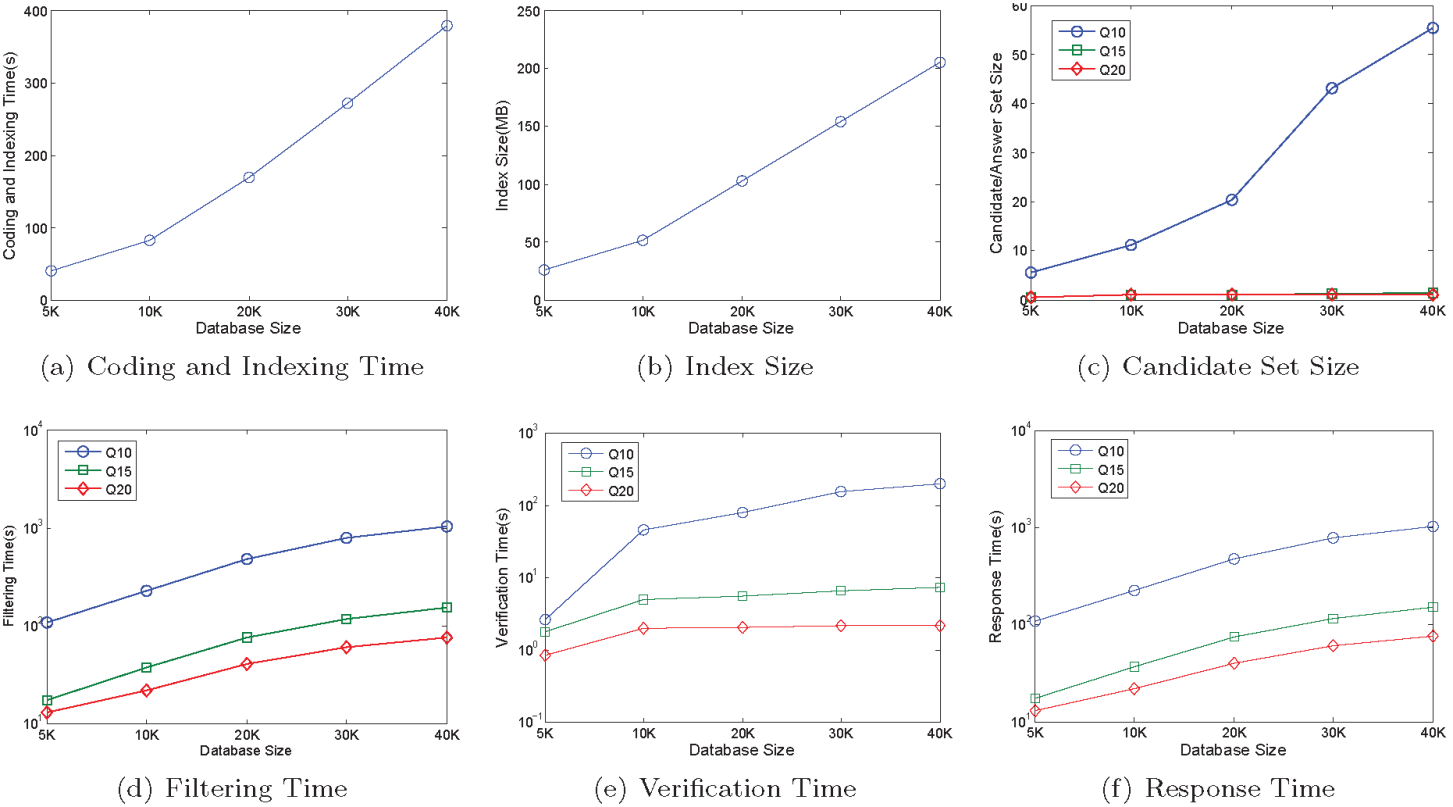
Performance on Graphs with Varying Sizes. (a) Coding and Indexing Time; (b) Index Size; (c) Candidate Set Size; (d) Filtering Time; (e) Verification Time; (f) Responde Time.

Fig. 17 shows the performance of our method on graphs with varying sizes. From it we observe that, with the increase of database size, the coding and indexing time and index size are almost linearly increasing. However, increasing rates of the candidate set size, the filtering time, the verification time, and the response time are much smaller except for the query graph set 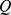
10, since its candidate set size grows much faster than those of 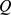
15 and 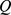
20. This indicates our method performs well on databases with different sizes.

#### Performance on graphs with varying vertex labels

In this experiment, we also generated five databases *D10L*, *D20L*, *D30L*, *D40L*, *D50L* by varying the vertex label. For database *DnL* (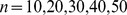), the number of vertex labels is *n*. The query graph sets are *Q*10, *Q*15 and *Q*20, where each query graph set *Qi* consists of 1,000 query graphs with *i* edges.

**Figure 18:**
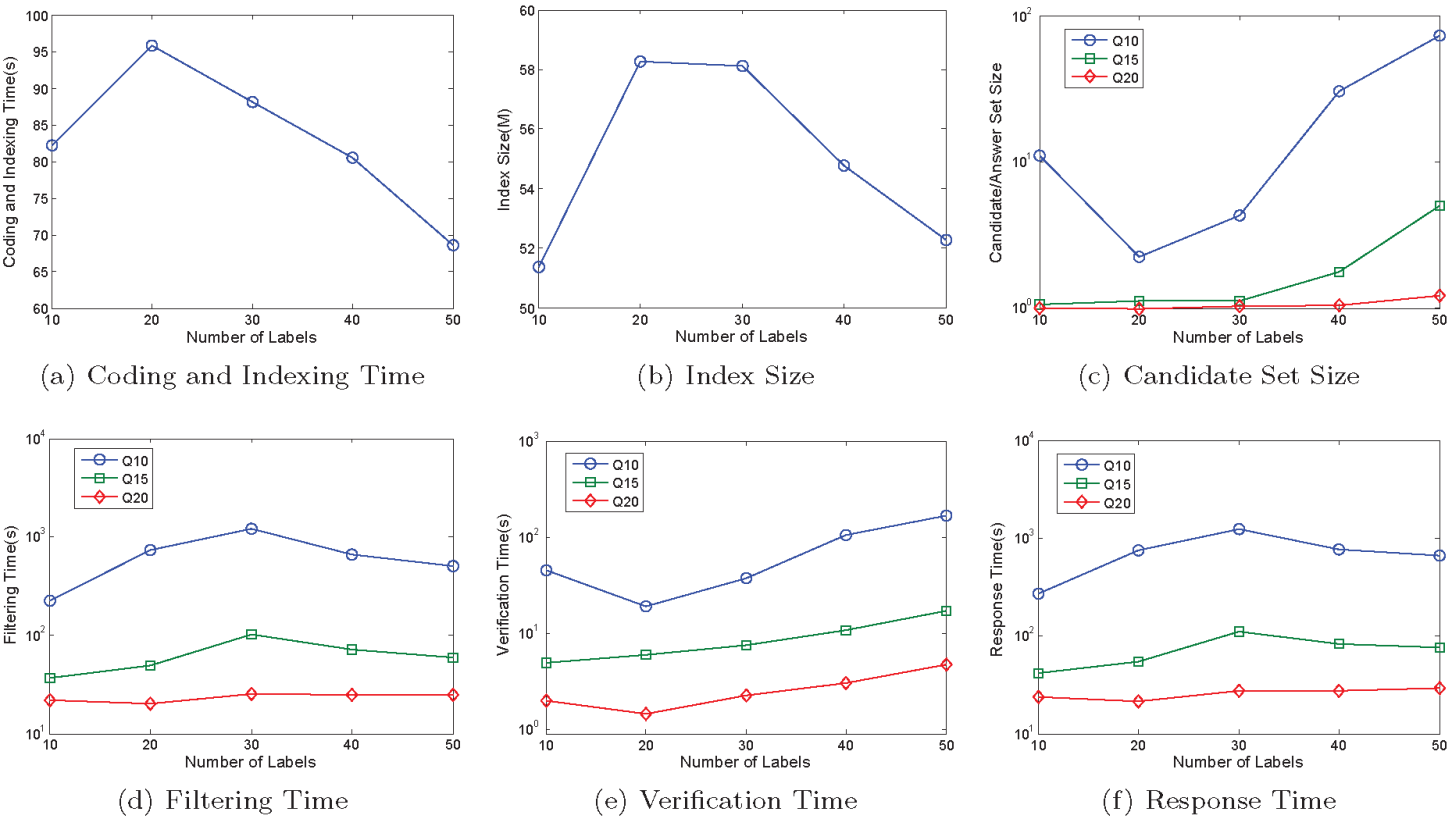
Performance on Graphs with Varying Vertex Labels. (a) Coding and Indexing Time; (b) Index Size; (c) Candidate Set Size; (d) Filtering Time; (e) Verification Time; (f) Responde Time.

Fig. 18 shows performance of our method on graphs with varying vertex labels. From it we know that, with the increase of the number of labels, 1) the coding and indexing time and the index size are decreasing except for the graphs with 10 labels, 2) the trends of the candidate set size, the filtering time, the verification time, and the response time are increasing but the growth rates are small or very small. This means our method works well on the graphs with varying vertex labels.

## Conclusions

In this paper, we propose a novel graph coding method LnGCoding, which utilizes the combination of Laplacian spectrum and the number of walks for subgraph querying over labeled graphs.

Our method first extracts some new graph features, and then maps these features into the numerical space to generate the vertex and graph codes. A novel index is built to improve the filtering efficiency. We also present novel two-step filtering conditions taking the properties of graph features into account, and the correctness is proved.

In order to evaluate the performance, extensive experiments on both real and synthetic data have been conducted. Experimental results show that, compared with the other six methods, our method works very well, especially when graphs are dense.

In the future, we plan using our graph coding method to explore similarity graph querying and supergraph querying.
